# A systems biology framework integrating GWAS and RNA-seq to shed light on the molecular basis of sperm quality in swine

**DOI:** 10.1186/s12711-020-00592-0

**Published:** 2020-12-08

**Authors:** Marta Gòdia, Antonio Reverter, Rayner González-Prendes, Yuliaxis Ramayo-Caldas, Anna Castelló, Joan-Enric Rodríguez-Gil, Armand Sánchez, Alex Clop

**Affiliations:** 1 Animal Genomics Group, Centre for Research in Agricultural Genomics (CRAG) CSIC-IRTA-UAB-UB, Campus UAB, Cerdanyola del Vallès, 08193 Barcelona, Catalonia Spain; 2 CSIRO Agriculture and Food, Queensland Bioscience Precinct, 306 Carmody Rd., St. Lucia, Brisbane, QLD 4067 Australia; 3 Animal Breeding and Genomics, Wageningen University & Research, 6708PB Wageningen, The Netherlands; 4 Animal Breeding and Genetics Program, Institute for Research and Technology in Food and Agriculture (IRTA), Torre Marimon, 08140 Caldes de Montbui, Catalonia Spain; 5 Unit of Animal Science, Department of Animal and Food Science, Autonomous University of Barcelona, Cerdanyola del Vallès, 08193 Barcelona, Catalonia Spain; 6 Unit of Animal Reproduction, Department of Animal Medicine and Surgery, Autonomous University of Barcelona, Cerdanyola del Vallès, 08193 Barcelona, Catalonia Spain; 7 Consejo Superior de Investigaciones Científicas (CSIC), 08003 Barcelona, Catalonia Spain

## Abstract

**Background:**

Genetic pressure in animal breeding is sparking the interest of breeders for selecting elite boars with higher sperm quality to optimize ejaculate doses and fertility rates. However, the molecular basis of sperm quality is not yet fully understood. Our aim was to identify candidate genes, pathways and DNA variants associated to sperm quality in swine by analysing 25 sperm-related phenotypes and integrating genome-wide association studies (GWAS) and RNA-seq under a systems biology framework.

**Results:**

By GWAS, we identified 12 quantitative trait loci (QTL) associated to the percentage of head and neck abnormalities, abnormal acrosomes and motile spermatozoa. Candidate genes included *CHD2*, *KATNAL2*, *SLC14A2* and *ABCA1.* By RNA-seq, we identified a wide repertoire of mRNAs (e.g. *PRM1*, *OAZ3*, *DNAJB8*, *TPPP2* and *TNP1*) and miRNAs (e.g. ssc-miR-30d, ssc-miR-34c, ssc-miR-30c-5p, ssc-miR-191, members of the let-7 family and ssc-miR-425-5p) with functions related to sperm biology. We detected 6128 significant correlations (P-value ≤ 0.05) between sperm traits and mRNA abundances. By expression (e)GWAS, we identified three trans-expression QTL involving the genes *IQCJ*, *ACTR2* and *HARS*. Using the GWAS and RNA-seq data, we built a gene interaction network. We considered that the genes and interactions that were present in both the GWAS and RNA-seq networks had a higher probability of being actually involved in sperm quality and used them to build a robust gene interaction network. In addition, in the final network we included genes with RNA abundances correlated with more than four semen traits and miRNAs interacting with the genes on the network. The final network was enriched for genes involved in gamete generation and development, meiotic cell cycle, DNA repair or embryo implantation. Finally, we designed a panel of 73 SNPs based on the GWAS, eGWAS and final network data, that explains between 5% (for sperm cell concentration) and 36% (for percentage of neck abnormalities) of the phenotypic variance of the sperm traits.

**Conclusions:**

By applying a systems biology approach, we identified genes that potentially affect sperm quality and constructed a SNP panel that explains a substantial part of the phenotypic variance for semen quality in our study and that should be tested in other swine populations to evaluate its relevance for the pig breeding sector.

## Background

Sperm carries the paternal genome and a wide repertoire of molecules including RNAs, which are essential for fertilization and the development of a new organism. Spermatogenesis, the process whereby germ cells proliferate and develop into mature spermatozoa, is controlled by multiple factors. Both DNA polymorphisms and gene expression have been linked to sperm quality and/or fertility in several mammalian species including cattle [1] and swine [2] and review in [3]. High-quality sperm is decisive to optimize the propagation of the best genetic material in livestock and the sustainability of the pig breeding sector. For this reason, ejaculated sperm is subjected to strict quality filters in boar artificial insemination (AI) studs. AI farms regularly evaluate the quality of ejaculates by measuring traits such as concentration, morphology, viability and motility kinetics, as a way to predict their fertilizing ability [4]. Although the heritability of these traits is low to moderate [5–8], the molecular processes and genetic mechanisms that control sperm quality are not yet fully understood and boar replacement due to insufficient sperm quality remains an economic hurdle for the sector [9].

Currently, there are few genetic and transcriptomic studies that have used high-throughput techniques to investigate the genetic basis of sperm quality in swine. To date, five genome-wide association studies (GWAS) have been performed. Diniz et al. [5] identified a single quantitative trait locus (QTL) region associated to sperm motility in Large White pigs. Two years later, Zhao and collaborators [10] reported three multi-single nucleotide polymorphism (SNP) QTL regions associated with epididymal weight, sperm concentration and total sperm per ejaculate, respectively and seven singleton QTL related to sperm motility, semen temperature, seminiferous tubule diameter and number of ejaculates in a White Duroc $\times$ Erhualian F_2_ population. Marques et al. [6] detected 16 and six QTL in Large White and Landrace pigs, respectively, associated with sperm motility, number of cells per ejaculate and morphological abnormalities. More recently, several QTL have been identified in a Duroc population associated to number of sperm cells, sperm motility, sperm progressive motility, total morphological abnormalities, coiled tail, bent tail, proximal droplets, distal droplets and distal midpiece reflex [11, 12].

The presence of RNA molecules in the boar sperm is well documented [13, 14], but their relation to sperm quality is very little explored. Porcine sperm RNAs are highly fragmented and their gene abundances are mostly associated to prior transcriptional events linked to spermatogenesis, fertility and embryo development [13]. A complex suite of RNAs are present in sperm, including coding (mRNA), long noncoding RNAs (e.g. circular RNA—circRNA-) and short noncoding RNAs (e.g. microRNA –miRNA- or Piwi interacting RNA—piRNA-) [13]. Several studies have reported a relation between RNA abundances and semen quality in mammals [15–17]. In swine, Curry et al. [18] performed quantitative RT-PCR (RT-qPCR) that targeted 10 miRNAs and identified five and two miRNAs associated to sperm morphology and motility, respectively. Moreover, our group has also identified a correlation between the abundance of some circRNAs [19] and piRNAs [20] with semen quality parameters in swine.

Based on these recent studies, it is now clear that the genetic complexity of sperm quality involves several molecular mechanisms and pathways that are highly interconnected. Complex traits are typically affected by a large number of genomic regions, many of which may explain only a small proportion of the phenotypic variance and do not reach significant levels in a GWAS or differential expression analysis. Moreover, classical GWAS or differential expression analyses carried out on a single trait [21] cannot consider the pleiotropic effects of variants or the interactions between them. In recent years, different methods such as the associated weight matrix (AWM) [22] and partial correlation coefficient with information theory (PCIT) [23] have been developed to carry out analysis of gene networks from GWAS or transcriptomics data and to identify co-associated genes for a set of correlated phenotypes [22, 24–26]. Furthermore, the integration of GWAS and RNA-seq data can be used to design knowledge-based technologies such as DNA marker panels including SNPs with a high functional potential for their application to animal breeding [27, 28]. SNPs that display a genetic association with a phenotype and show functional potential (e.g. coding or regulatory variants) are less likely to show spurious associations than non-functional SNPs.

Our aim was to identify candidate genes, pathways and DNA variants associated to sperm quality in pigs by integrating GWAS and RNA-seq results under an unprecedented systems biology approach. Moreover, we sought to estimate the weight of the most relevant genes and DNA variants on the sperm phenotypes.

## Methods

### Sample collection and phenotype measurements

Three hundred fresh sperm ejaculates, each from a different Pietrain boar from commercial farms, were collected by specialized professionals between September 2014 and January 2017. Sperm was obtained using the gloved-hand method [29], immediately diluted (1:2) in commercial extender and kept at 16°C for up to 2 h until phenotype assessment. Blood samples were collected from specialists during their routine sample collection and gDNA was extracted using a phenol–chloroform based method [30]. The ejaculates were purified to remove somatic cells as described previously [14] and purified spermatozoa were stored with Trizol® at − 80°C until further use.

Phenotypic records from fresh sperm were measured as previously described [14] and included: sperm concentration (CON), percentage of viable cells (VIAB), percentage of morphologically abnormal acrosomes (ACRO), osmotic resistance test (ORT), percentage of morphologically abnormal sperm cells (of the head -HABN-, neck -NABN- and tail -TABN-) and of cells with cytoplasmatic droplets (proximal -PDROP- and distal -DDROP-). Sperm motility traits were also assessed using the computer-assisted semen analysis (CASA) system (Integrated Sperm Analysis System V1.0; Proiser) and included the percentage of motile spermatozoa cells (MT) (with average path velocity -VAP- > 10 µm/s), average curvilinear velocity (VCL) (µm/s), average straight-line velocity (VSL) (µm/s) and average VAP (µm/s). All phenotypes were assessed after 5 and 90 min of incubation of the samples at 37°C, except for sperm concentration, ORT, sperm abnormalities and cytoplasmatic droplets, which were measured only after 5 min of incubation at 37°C. To calculate the correlations between RNA abundance and phenotype, sperm traits were corrected using the fixed effects of farm of origin (3 levels), season and year of collection (9 levels) and boar age (3 levels) with the "lm" function of R [31] using a linear model. The 90 min/5 min incubation ratios were also calculated. In total, 25 phenotypic measures per sample were recorded. Phenotypic correlations between traits were assessed and graphically displayed with the R package “corrplot” [32].

The different analyses are described below, and the complete outline is summarized in Additional file 1: Figure S1.

### Genome-wide association study (GWAS)

Two hundred and eighty-eight boars were genotyped using the high-density (660 K markers) Axiom™ Porcine Genotyping Array (Thermo Fisher Scientific). The resulting genotype dataset was stringently filtered by excluding the samples with a genotype call rate lower than 96%. SNP locations were converted from Sscrofa10.2 to Sscrofa11.1 coordinates using plink v1.9 [33]. Then, we excluded SNPs that (i) had a minor allele frequency lower than 0.05, (ii) deviated from Hardy–Weinberg equilibrium (P-value ≤ 0.001), and (iii) for which there were more than 5% missing genotypes. These are standard parameters that are typically used in similar studies [34–36]. Single-SNP association analysis was carried out using the genome-wide complex trait analysis (GCTA) v.1.91.5 software [37] with the following model:

$$Y_{ijklm} = \mu + \delta SNP_{i} + Farm_{j} + SeasonYear_{k} + Age_{l} + u_{m} + e_{ijklm},$$

where ${Y}_{ijkl}$ is the phenotype modeled as a function of the population mean ($\upmu$), $\delta$ is the SNP allelic effect, estimated as a regression coefficient on the corresponding (values − 1, 0, 1) of the SNP $i$; correcting for the fixed effect of farm (${\mathrm{Farm}}_{\mathrm{j}}$), season and year ${(SeasonYear}_{k})$ and boar age (${Age}_{l}$); ${u}_{m}$ is the infinitesimal genetic effect of individual $m$, with $u\sim N\left(0,\mathbf{G}{\sigma }_{u}^{2}\right)$, where $\mathbf{G}$ is the genomic relationship matrix (GRM) calculated using the filtered SNPs based on the methodology described by Yang et al. [37], and ${\sigma }_{u}^{2}$ is the additive genetic variance; and ${e}_{ijklm}$ is the residual term.

The significance of SNP associations was corrected for multiple testing with the false discovery rate (FDR) approach [38] and only significant SNPs (FDR ≤ 0.05) were kept for further analysis. Significantly associated SNPs with consecutive distances shorter than 5 Mbp were considered to belong to the same GWAS interval [39]. A new interval was called if the consecutive SNPs were more than 5 Mbp apart. SNPs that mapped to the sex chromosomes or to unmapped scaffolds were not considered for further analysis. Genomic heritability was assessed with GCTA v.1.91.5 through a genomic restricted maximum likelihood (GREML) approach using the GRM based on the methodology from Yang et al. [37]. Manhattan plots of the GWAS results displaying the genetic associations (P-value) between each SNP and phenotype were generated with the “qqman” R package [40].

### RNA isolation, sequencing and gene annotation

RNA isolation from 40 sperm samples was performed as previously described [14] and included 35 samples from boars analyzed in the GWAS. The other five boars did not pass the genotyping quality control and thus were not included in the GWAS. Extracted RNA was subjected to quality control assays including quantification with the Qubit™ RNA HS Assay kit (Invitrogen), assessment of RNA integrity with the 2100 Bioanalyzer using the Agilent RNA 6000 Pico kit (Agilent Technologies), and evaluation by RT-qPCR of the sperm-specific *PRM1*, the somatic *PTPRC* mRNA and genomic DNA to confirm that the samples were free from somatic cell RNA and gDNA contaminations.

The ribosomal RNA (rRNA) from the 40 RNA samples was depleted with the Ribosomal RNA depletion Kit (Illumina) and libraries were prepared with the SMARTer Low Input Library Prep kit v2 (Clontech) and sequenced to generate 75 bp pair-end reads on an Illumina’s HiSeq2000/2500. Undepleted total RNA was also subjected to short noncoding RNA (sncRNA) library preparation (34 of the previous 40 samples) using the NEBNext library prep kit (New England Biolabs) and sequenced at 50 bp single-end on a Hiseq2000 (Illumina).

Total RNA-seq reads were evaluated for quality control with the FastQC software (https://www.bioinformatics.babraham.ac.uk/projects/fastqc/). Low-quality reads (phred –Q < 20 and read length < 25 bp) and sequencing adaptors were trimmed with Trimmomatic v.0.36 [41]. Filtered reads were mapped to the porcine genome (Sscrofa 11.1) using HISAT2 v.2.1.0 [42]. Duplicate reads were removed with Picard Tools v.2.18.29 (http://picard.sourceforge.net) Markduplicates. RNA levels of the genes annotated in the porcine genome (Ensembl v.91) were then quantified with StringTie v.1.3.4 [43]. Only the genes with average RNA abundances ≥ 10 fragments per kb of exon per million reads mapped (FPKM) were kept for further analysis with the aim to discard low abundant genes and spuriously mapped reads.

The effect of external variables on gene expression was assessed using the following mixed effect model as in Reverter et al. [44]:

$$Y_{ijklmn} = \mu + L_{i} + G_{j} + GF_{jk} + GYS_{jl} + GA_{jm} + GR_{jn} + e_{ijklmn},$$

where ${Y}_{ijklmn}$ represents the log2-transformed FPKM value from library $i$ (40 levels), gene $j$ (4120 levels), farm $k$ (3 levels), year-season $l$ (6 levels), age $m$ (3 levels) and assay run $n$ (4 levels). Accordingly, ${Y}_{ijklmn}$ was modeled as a function of the mean *(*$\mu$), fixed effect of library (${L}_{i}$) and the random effects of gene (${G}_{j}$), gene by farm (${GF}_{jk}$), gene by year-season (${GYS}_{jl}$), gene by age (${GA}_{jm}$) and gene by assay run (${GR}_{jn}$). Random residuals in ${e}_{ijklmn}$ were assumed to be independent and identically distributed. Using standard stochastic assumptions, the effects of ${G}_{j}$, ${GF}_{jk}$, ${GYS}_{jl}$, ${GA}_{jm}$ and ${GR}_{jn}$ were assumed to follow a normal distribution with zero mean and between-gene, between-gene within-farm, between-gene within year-season, between-gene within age, between-gene within assay and within gene components of variance, respectively. Restricted maximum likelihood estimates and solutions to model effects were obtained using VCE6 [45].

For the sncRNA-seq data, trimming of adaptors and low-quality bases (phred –Q < 20 and read length < 12 bp) was performed with Cutadapt v1.0 [46]. Reads were mapped to the *Sus scrofa* genome (Sscrofa11.1) with the sRNAtoolbox v.6.17 [47] using default settings and with the porcine miRBase [48] release 21 database. Multi-adjusted read counts were normalized by library size as counts per million (CPM). Only miRNAs with an average abundance higher than 1 CPM in all the samples were considered. miRNA abundance was stabilized with the log2 transformation.

The relationship between the 25 phenotypes and each of the log2-stabilized mRNA’s and miRNA's abundances were calculated using the Pearson correlation coefficient. Only correlations with a P-value ≤ 0.05 were kept.

### SNP calling from RNA-seq data and linkage disequilibrium with GWAS lead SNPs

Mapped RNA-seq reads of the 35 samples with RNA-seq and genotype data were subjected to SNP calling. Variant calling was performed with SAMtools mpileup and BCFtools v.1.9 [49]. Only SNP variants for which the alternative allele was present in at least 10 samples with a minimum Phred quality of 25 and a minimum read depth of 10 were kept. The effect of the SNP on protein sequence was predicted with SnpEff v.4.3T [50] and only low, moderate and high impact variants were kept. The new SNP genotypes were merged to the Axiom genotypes and the linkage disequilibrium (LD) R^2^ between GWAS lead SNPs and RNA-seq SNPs was assessed with PLINK v1.9 [33] using the default parameters, with the exception of “-ld-window 100”, “-ld-window-kb 0” and “–ld-window-r2 0” to assess all the pair-wise LD values.

### Expression GWAS

Expression GWAS (eGWAS) included the 35 samples with RNA-seq and genotype data. The RNA abundances of the detected genes were taken as quantitative traits and tested for association with the genotypes that passed quality control using a linear model. Single-SNP association analysis was performed with the GCTA v.1.91.5 software [37], with the following model:

$$Y_{i} = \mu + SNP_{i} + e_{i},$$

where ${Y}_{i}$ is the log2-transformed gene abundance modeled as a function of the population mean ($\mu$), fixed effect of each SNP (${SNP}_{i}$), and a residual effect (${e}_{i}$).

eGWAS significant associations (FDR ≤ 0.05) were considered only if: (i) the eGWAS associated SNP was also a significant hit (FDR ≤ 0.05) in the GWAS for sperm quality phenotypes and (ii) the gene’s RNA abundance correlated to the same phenotype as the corresponding GWAS SNP hit.

### SNP co-association and gene co-abundance analyses

We also carried out a SNP co-association analysis by building an AWM from the GWAS results [22, 51]. The AWM was constructed from two matrices that contained row-wise SNPs and column-wise phenotypes. The first matrix included the P-values of the association between each SNP and the phenotype, and the second matrix corresponded to the SNP z-score standardized additive effect. As live cells with intact plasma membrane are essential for fertilization [52, 53], the percentage of viable spermatozoa at 5 min (VIAB_5) was selected as key phenotype and the associated SNPs (P-value ≤ 0.01) were included in the AWM. In the next step, the dependency between phenotypes was estimated based on the average number of non-key phenotypes associated (Ap) with these SNPs (P-value ≤ 0.01) (Ap ≥ 2). Then, SNPs that were located less than 2500 bp or more than 1 Mbp from the nearest annotated gene (Ensembl v.91) were kept. The most significant SNP from each annotated gene was kept to build the AWM. The standardized SNP effects across phenotypes were computed and represented using the hierarchical cluster analysis based on Euclidean distance with the R package “dendextend” [54]. Then, significant gene–gene interactions were assessed to build the SNP network with the PCIT algorithm [23]. PCIT applies first-order partial correlation coefficients together with an information theory approach to identify meaningful gene–gene associations [23]. Only significant gene co-associations (P-value ≤ 0.05) were kept in the SNP network.

For the RNA co-abundance analysis, significant gene–gene interactions that were used to build the RNA network were also predicted with PCIT using the stabilized RNA abundances. Interactions between genes and miRNAs were also assessed with PCIT [23], and only significant negative correlations (P-value ≤ 0.05) were kept.

### Integration of SNPs and RNA network data and network visualization

The genes and interactions that were present in both the GWAS and RNA-seq networks were considered to have a higher probability of being involved in sperm quality and were used to build a robust gene interaction network. The resulting network was named “shared network”. In addition, the genes that were not present in the shared network but that presented an abundance correlation with more than three phenotypes and their co-associated genes were merged with the shared network to create the so-called final network. This final network also included the interactions between miRNA and mRNA genes. Network visualization was performed with Cytoscape v3.6 [55] and included information on: (i) the number of phenotypes associated to a gene or miRNA, (ii) the phenotype with the highest correlation for each gene, (iii) whether the gene was annotated as a transcription factor (TF) or TF co-factor, and (iv) whether the gene was present in the shared network or was only found in the final network. TF and TF co-factors were extracted from the AnimalTFDB3.0 database [56].

### Development of an RNA model and SNP panel for the phenotypic prediction of sperm quality

The unadjusted RNA abundance of a subset of the genes in the network was used to identify which combination of genes was a better predictor of sperm quality phenotypes. For this, first we extracted 20 genes from the network. These genes were (i) correlated with at least four phenotypes, (ii) did not present interactions (edges) between them, (iii) all samples presented RNA abundance levels higher than 0 FPKM, and (iv) were potentially relevant according to the existing literature. The RSQUARE statement of the REG procedure implemented in the SAS software [57] was used as an exploratory model to evaluate all possible subsets of linear regressions using unadjusted gene abundances and sperm phenotypes and extract the R^2^ magnitude from each prediction. Then, we selected the subset of 10 genes that were most commonly present in all the phenotype models. This subset of common genes was then used for the STEPWISE statement of the REG procedure implemented in the SAS software [57], which performs a linear regression analysis for each of the phenotypes to develop a model to predict the phenotype based on gene RNA levels. The model is:

$$Y_{ij} = intercept_{i} + GPE_{ij} + e_{ij},$$

where $${Y}_{ij}$$ represents the predicted phenotype value from $$i$$-th phenotypes (25 levels), $$j$$-th genes (10 levels). $${Y}_{ij}$$ was modeled as a function of the intercept value for the phenotype ($${intercept}_{i})$$, the gene abundance by parameter estimate ($$G{PE}_{ij})$$ and a residual term ($${e}_{ij})$$. The accuracy was ascertained from the model’s goodness-of-fit and based on the proportion of variance explained by the model (R^2^). We also developed a genome-wide SNP panel to identify the SNPs that could best predict the phenotypic variance of sperm-related traits. The panel included the lead SNPs from the GWAS and from the eGWAS hits, and the GWAS most significant SNP for each of the genes included in the network that also: (i) correlated with at least four phenotypes and (ii) were identified in the shared network. The proportion of the phenotypic variance explained by these SNPs was estimated with the GREML analysis implemented in the GCTA software using the GRM calculated with the 73 autosomal SNPs based on the methodology from Yang et al. [37].

## Results

### Phenotypic parameters

Three hundred ejaculates were phenotyped for 25 sperm quality traits (Table 1). Phenotype correlations (see Additional file 2: Figure S2) were consistent with their physiological similarities. In general, SNP-based heritabilities (Table 1) were low to moderate with motility-related traits displaying higher values. MT_90 was the most heritable trait (h^2^: 0.39), whereas motility ratios, NABN and VIAB_5 showed heritability values close to 0 (Table 1). The sperm phenotypes correlated with farm, boar age and season per year (see Additional file 3: Table S1) and were thus included as fixed effects in the GWAS model and phenotypes were also corrected for these effects to carry out the correlation analysis.

**Table 1:** Descriptive statistics, genomic heritability (h^2^) and number of significant SNPs in the GWAS for sperm quality parameters (N = 300)

Trait	Acronym	Mean (SD)	h^2^ (SE)	Number of SNPs in autosomal chromosomes	Number of SNPs in unplaced scaffolds
Concentration (sperm/mL)	CON	141.3 (65.5)	0.13 (0.11)	0	0
Viability 5 min	VIAB_5	90.1 (6.3)	1 × 10^–6^ (0.11)	0	0
Viability 90 min	VIAB_90	77.4 (17.3)	0.14 (0.13)	0	0
Osmotic resistance test	ORT	79.8 (12.5)	0.13 (0.12)	0	0
Head abnormalities	HABN	2.1 (5.9)	0.16 (0.11)	41	0
Neck abnormalities	NABN	3.0 (4.9)	1 × 10^–6^ (0.13)	18	0
Tail abnormalities	TABN	2.7 (3.4)	0.09 (0.12)	0	0
Proximal droplets	PDROP	3.5 (5.1)	0.12 (0.15)	1	0
Distal droplets	DDROP	4.5 (4.5)	0.06 (0.11)	0	0
Motility 5 min	MT_5	75.4 (18.1)	0.21 (0.15)	3	217
Motility 90 min	MT_90	64.1 (22.0)	0.39 (0.14)	2	252
Average path velocity 5 min (µm/seg)	VAP_5	34.0 (10.2)	0.17 (0.11)	0	0
Average path velocity 90 min (µm/seg)	VAP_90	30.8 (9.5)	0.35 (0.13)	0	0
Curvilinear velocity 5 min (µm/seg)	VCL_5	46.2 (12.5)	0.11 (0.10)	0	0
Curvilinear velocity 90 min (µm/seg)	VCL_90	39.7 (10.2)	0.35 (0.13)	0	0
Straight line velocity 5 min (µm/seg)	VSL_5	27.0 (8.3)	0.23 (0.13)	0	38
Straight line Velocity 90 min (µm/seg)	VSL_90	25.9 (8.3)	0.34 (0.13)	0	0
Abnormal acrosomes 5 min	ACRO_5	7.0 (5.6)	0.08 (0.11)	4	0
Abnormal acrosomes 90 min	ACRO_90	16.4 (12.6)	0.06 (0.10)	0	0
Ratio motility	R_MT	0.9 (0.2)	1 × 10^–6^ (0.11)	0	0
Ratio average path velocity	R_VAP	0.9 (0.3)	1 × 10^–6^ (0.08)	0	0
Ratio Curvilinear velocity	R_VCL	0.9 (0.3)	1 × 10^–6^ (0.09)	0	0
Ratio straight line velocity	R_VSL	1.0 (0.3)	0.06 (0.10)	0	0
Ratio viability	R_VIAB	0.9 (0.3)	0.08 (0.11)	0	0
Ratio acrosomes	R_ACRO	3.4 (3.5)	0.08 (0.11)	1	0

All traits except stated are presented as a percentage

Number of SNPs = GWAS number of single nucleotide polymorphisms significantly associated (FDR) with the trait

The values shown are raw excepting the ratios which were previously corrected and stabilized

*SD* standard deviation, *SE* standard error

### GWAS analysis

After quality control, 466,592 SNPs and 276 samples remained for the GWAS. In total, 324 SNPs across the autosomal chromosomes and unplaced scaffolds displayed genetic associations (FDR ≤ 0.05) with one or more sperm quality phenotype (Table 1) and (see Additional file 4: Table S2). Among these 324 SNPs, 255 mapped to unplaced scaffolds and were not considered for further data analysis (Additional file 4: Table S2). Nineteen chromosomal regions tagged by 69 significant SNPs were identified on *Sus scrofa* (SSC) chromosomes 1, 3, 4, 6, 7, 9, 13 and 16. The number of SNPs that displayed significant associations (FDR ≤ 0.05) for each trait is summarized in Table 2.

**Table 2:** Summary of the results of the genome wide association analysis for sperm quality traits

SSC	Interval	#SNP	Interval Mbp	Top SNP	Top SNP location bp	Top SNP P-value	Top SNP FDR	Top SNP MAF	Beta	Trait
1	I1	1	–	rs339761632	13,501,755	4.64 × 10^–8^	0.02	0.06	4.84	PDROP
1	I2	8	82.90–83.49	rs81354986	82,895,619	1.69 × 10^–6^	0.03	0.07	5.05	HABN
1	I3	8	94.88–98.74	rs327733412	94,880,167	1.61 × 10^–7^	0.02	0.07	5.65	HABN
1	I4	1	–	rs337166779	126,397,198	2.05 × 10^–6^	0.03	0.06	5.02	HABN
1	I5	11	243.86–246.44	rs343194423	246,224,386	1.72 × 10^–7^	0.01	0.07	3.17	NABN
1	I6	2	258.54–258.55	rs332256425	258,548,786	1.76 × 10^–6^	0.04	0.06	3.44	NABN
3	I1	1	–	rs332055717	2,911,413	6.35 × 10^–8^	0.01	0.09	5.07	HABN
3	I2	3	113.75–113.84	rs328292697	113,750,595	1.09 × 10^–7^	0.01	0.07	3.41	NABN
4	I1	2	2.41–2.42	rs318575212	2,412,006	2.88 × 10^–8^	0.01	0.08	4.11	ACRO_5
				rs332927981	2,415,239					
6	I1	2	65.60–66.66	rs335394654	65,597,553	1.86 × 10^–7^	0.03	0.14	3.04	ACRO_5
7	I1	2	6.20–6.38	rs326239534	6,377,172	9.87 × 10^–6^	0.02	0.17	-9.15	MT_5
7	I2	2	85.73–86.88	rs336588919	86,884,279	4.13 × 10^–8^	0.01	0.06	3.75	NABN
9	I1	2	5.76–5.78	rs1110111787	5,776,597	1.55 × 10^–7^	0.02	0.07	5.43	HABN
9	I2	1	–	rs342738178	28,463,580	1.53 × 10^–5^	0.03	0.14	− 10.42	MT_5, MT_90
9	I3	1	–	rs328217450	137,959,590	4.77 × 10^–8^	0.02	0.18	2.36	R_ACRO
13	I1	18	25.36–28.47	rs690794887	25,535,100	3.06 × 10^–7^	0.02	0.14	3.78	HABN
13	I2	3	33.82–37.65	rs327865244	33,819,549	3.79 × 10^–8^	0.01	0.15	4.28	HABN
16	I1	1	–	rs324239602	6,476,358	6.08 × 10^–6^	0.01	0.46	9.07	MT_90

SSC, *Sus scrofa* chromosome; #SNP, number of SNPs significantly associated (FDR) with the trait; Interval, region of the GWAS interval; Beta, additive effect; FDR, false discovery rate; MAF, minor allele frequency; ACRO_5, abnormal acrosomes 5 min; HABN, head abnormalities; NABN, neck abnormalities; PDROP, proximal droplets; R_ACRO, ratio acrosomes; MT_5, motility 5 min; MT_90, motility 90 min

**Fig. 1:**
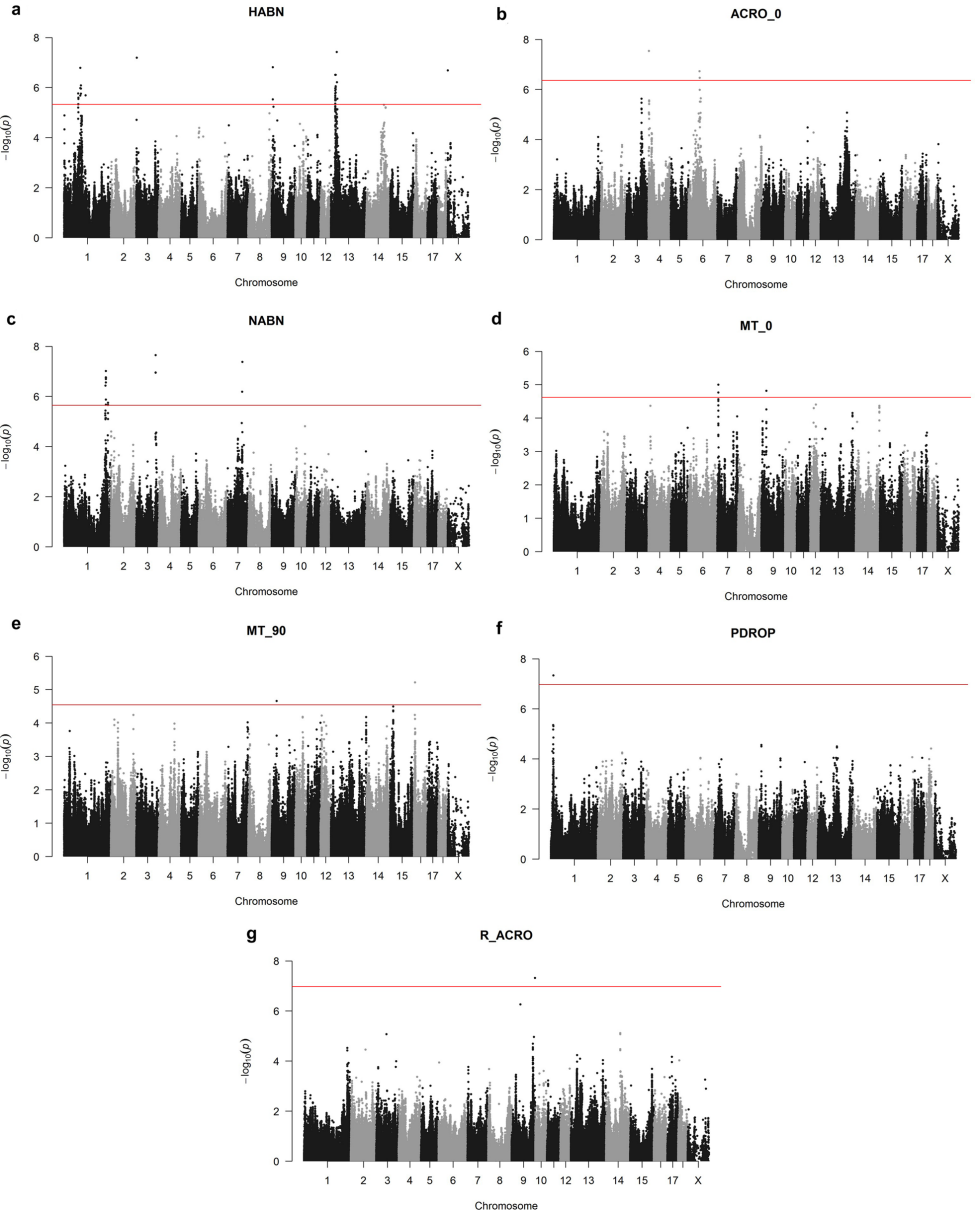
Manhattan plots depicting the genetic associations between SNPs and the sperm quality traits that showed genome-wide significant values. Significant associations have been found with the percentage of: **a** Percentage of cells with head abnormalities (HABN); **b** percentage of cells with abnormal acrosomes after 5 min incubation at 37°C (ACRO_5); **c** percentage of cells with neck abnormalities (NABN); **d** percentage of motile spermatozoa after 5 min incubation at 37°C (MT_5); **e** Percentage of motile spermatozoa after 90 min incubation at 37°C (MT_90); **f** Percentage of cells with proximal droplets (PDROP); **g** Ratio of the percentage of abnormal acrosomes at 5 min versus 90 min incubation times (R_ACRO). The *x*-axis represents chromosome length (Mb), and the *y*-axis shows the negative log_10_ P-values of the genetic associations. The horizontal red line represents the significance threshold (FDR ≤ 0.05)

Seven sperm quality traits exhibited significant association signals (Fig. 1a–g) and (see Additional file 4: Table S2), and only one SNP was associated with more than one trait (Table 2; Fig. 1d, e) and (see Additional file 4: Table S2). The number of SNP signals was largest for HABN and NABN with 41 and 18 associated SNPs, respectively (Fig. 1a, c) and (see Additional file 4: Table S2). Six of the 19 QTL were represented by one associated SNP only and were discarded from further analyses (Table 2; Fig. 1). The most significant SNPs (rs318575212 and rs332927981) were associated with ACRO_5 (both with FDR = 0.006 and an additive effect = 4.11) (Table 2).

### Sperm RNA isolation, RNA-seq and bioinformatics analysis

Isolated RNA from mature spermatozoa was free from somatic cell RNA. Total RNA-seq resulted in an average of 40.7 M reads per sample and 98.2% of the reads passed the quality control filters (see Additional file 5: Table S3). An average of 83% of the reads mapped to the porcine genome and after removal of duplicate reads and RNA abundance filters, we identified 4120 genes (see Additional file 6: Table S4). The most abundant protein coding transcripts included *PRM1*, *OAZ3*, *DNAJB8*, *ANKRD35*, *SPATC1* and *ZNRF4*, among others, as well as mitochondrial genes such as *COX1*, *CYTB* and *ND5* (see Additional file 6: Table S4). The variance component estimated by the mixed model explained 84% (80% due to the main effect of the gene) of the variation in gene abundance. Consequently, RNA abundances were not corrected for external effects. For short RNA-seq, we obtained an average of 7.3 M of reads per sample. Of these, 99.2% passed quality control and 81.5% mapped to the porcine genome (see Additional file 5: Table S3). In more detail, 42% of the aligned reads corresponded to sncRNAs, including piRNAs (16%), snRNA (8%), tRNA (9%) and miRNA (9%) (see Additional file 5: Table S3). The remaining aligned reads corresponded to mitochondrial transfer and ribosomal RNAs (see Additional file 5: Table S3). We identified 95 miRNAs out of the 306 that are annotated in swine (see Additional file 6: Table S4). The most abundant miRNAs with CPM higher than 1000 were ssc-miR-30d, ssc-miR-34c, ssc-miR-30c-5p, ssc-miR-191, ssc-let-7a, ssc-let-7g, ssc-miR-28-3p and ssc-miR-425-5p (see Additional file 6: Table S4).

### SNP calling from RNA-seq and linkage disequilibrium with GWAS hits

Under the hypothesis that some of the GWAS hits may tag a causal variant that alters the protein sequence and function, and to identify additional SNPs with the potential to obtain better genetic markers than those identified in the GWAS, we sought to identify variants in annotated genes using the RNA-seq data. As a prerequisite, these variants had to be in LD with the cognate GWAS hit. After filtering, we identified 7719 expressed variants, 37 of which were located within the genomic intervals identified in the GWAS (Table 2) and (see Additional file 7: Table S5). Twenty-three SNPs were predicted to have a low effect on protein sequence (synonymous variants and 5′ UTR premature start codon), 13 SNPs showed a moderate effect (missense variants) and one SNP was predicted as a splice donor variant and thus, to have a high impact on protein sequence (see Additional file 7: Table S5).

Interval 1 (I1) on SSC13 was associated to HABN, harboured 21 expressed SNPs (7 and 14 with moderate and low effects, respectively). The polymorphism rs331304027 (a missense variant with a moderate effect on the *ULK4* gene) was in LD (LD = 0.40) with the strongest GWAS SNP hit of the interval (rs690794887) (Table 3). SSC13 I2, was also associated to HABN, included 11 SNPs (1 with a high, 5 with a moderate and 5 with a low effect on protein sequence). Of these, the variant with the highest LD (LD = 0.2) with the GWAS hit (rs327865244) was a 5′ UTR premature start codon gain (low effect) SNP (rs323872641) in the *ABHD14A* gene (Table 3) and (see Additional file 7: Table S5). This interval was the only one that presented a SNP with a high effect (novel), a splice donor variant in the *IQCF5* gene, with almost no LD (LD = 0.02) with the GWAS hit (see Additional file 7: Table S5). SSC7 I2 was associated to NABN and encompassed two expressed SNPs (both with a low effect). rs330912302 (a synonymous SNP in the *CHD2* gene) presented an LD (LD = 0.4) with the strongest hit of the interval (rs336588919) (Table 3). SSC1 I3 was associated to HABN and harboured three expressed SNPs (1 with a moderate and two with a low effect) (Table 3) and (see Additional file 7: Table S5).

**Table 3:** Summary of the SNPs identified from the RNA-seq datasets in genes mapping within the GWAS regions

SSC	Interval	Top SNP of the GWAS interval	# SNPs called	Highest LD	SNP with highest LD	Genotypic frequency (0/0; 0/1; 1/1)	# called samples	SNP effect	Gene	Trait
1	I3	rs327733412	3	0.07	rs710447566	0.34; 0.54; 0.11	35	Low	*KATNAL2*	HABN
7	I2	rs336588919	2	0.4	rs330912302	0.63; 0.12; 0.25	32	Low	*CHD2*	NABN
13	I1	rs690794887	21	0.4	rs331304027	0.06; 0.09; 0.85	33	Moderate	*ULK4*	HABN
13	I2	rs327865244	11	0.2	rs323872641	0.49; 0.37; 0.14	35	Low	*ABHD14A*	HABN

SSC, *Sus scrofa* chromosome; # SNPs called, number of SNPs identified in the SNP calling analysis; LD, linkage disequilibrium; Genotypic frequency: allelic frequency for each of the genotypes; # called samples, number of samples with reads in the given SNP position; HABN, head abnormalities; NABN, neck abnormalities

The columns SNP effect and gene refer to the SNP with the highest LD in the region

### Correlation of genes’ and miRNAs’ abundances with sperm quality traits

The correlation analysis of the 4120 genes and the 25 phenotypes resulted in 6128 significant correlations (P-value ≤ 0.05) involving 3007 genes and the 25 traits (see Additional file 8: Table S6). These genes presented between one and nine significant correlations with the different semen quality traits (see Additional file 8: Table S6). Three hundred and forty-four genes were significantly correlated with more than four traits. For the miRNAs, the abundance of the 95 miRNAs and the studied phenotypes resulted in 306 significant correlations (P-value ≤ 0.05) which involved 87 miRNAs and 17 semen traits (see Additional file 9: Table S7). The miRNAs presented between 1 and 9 significant correlations with the semen quality traits (see Additional file 9: Table S7).

### Expression GWAS analysis

In order to predict whether the GWAS hits tagged a causal variant that altered gene expression, we performed a within-trait eGWAS with the genotypes of 464,020 SNPs that passed the quality control and the normalized RNA abundances. Then, we focused only on the associations between GWAS SNP hits (with FDR ≤ 0.05) and transcripts with abundances that correlated with the same phenotype. We identified 45 SNPs (FDR ≤ 0.05) that were located in three genomic regions related to ACRO_5 and HABN (Table 4). Six SNPs had unknown positions on the genome after the lift-over from Sscrofa10.2 to Sscrofa11.1. The remaining eGWAS hits were on SSC4, 6 and 13 (Table 4) and (see Additional file 10: Table S8). All the SNPs had a *trans* effect on genes that were located on other chromosomes. The eQTL identified on SSC4, was related to ACRO_5 and was associated to three genes, *NCLN*, *ASCC1* and *AATF*. The eQTL on SSC6 was also related to ACRO_5 and was associated to the *IQCJ* gene. Finally, the eQTL on SSC13 for HABN, included SNPs that were in the *HARS, ACTR2, EPB41L3* and *RAB1B* genes.

**Table 4:** Summary of the results from the within-trait expression genome wide association analysis

SSC	Interval	# SNP: transcripts	Top eGWAS	Top eGWAS location bp	Top eGWAS P-value	Top eGWAS FDR	Top eGWAS MAF	Beta	Trait	RNA abundance correlation	Associated gene
4	I1	2	rs318575212	2,412,006	7.36 × 10^–3^	0.03	0.09	− 0.39	ACRO_5	− 0.33	*NCLN*
			rs332927981	2,415,239							
		2	rs318575212	2,412,006	1.83 × 10^–4^	0.03	0.09	− 1.8	ACRO_5	− 0.46	*ASCC1*
			rs332927981	2,415,239							
		2	rs318575212	2,412,006	2.87 × 10^–4^	4.83 × 10^–2^	0.09	− 1.1	ACRO_5	− 0.4	*AATF*
			rs332927981	2,415,239							
6	I1	2	rs335394654	65,597,553	5.63 × 10^–5^	0.02	0.11	− 1.65	ACRO_5	− 0.35	*IQCJ*
13	I1	31	rs328397029	25,684,259	1.84 × 10^–5^	2.95 × 10^–3^	0.09	− 1.03	HABN	− 0.38	*HARS, ACTR2, EPB41L3*, *RAB1B*

SSC, *Sus scrofa* chromosome; # SNP: transcripts, number of single nucleotide polymorphisms significantly associated to a transcript; Beta, additive effect; MAF, minor allele frequency; ACRO_5, abnormal acrosomes 5 min; HABN, head abnormalities

### Gene network analysis

**Fig. 2:**
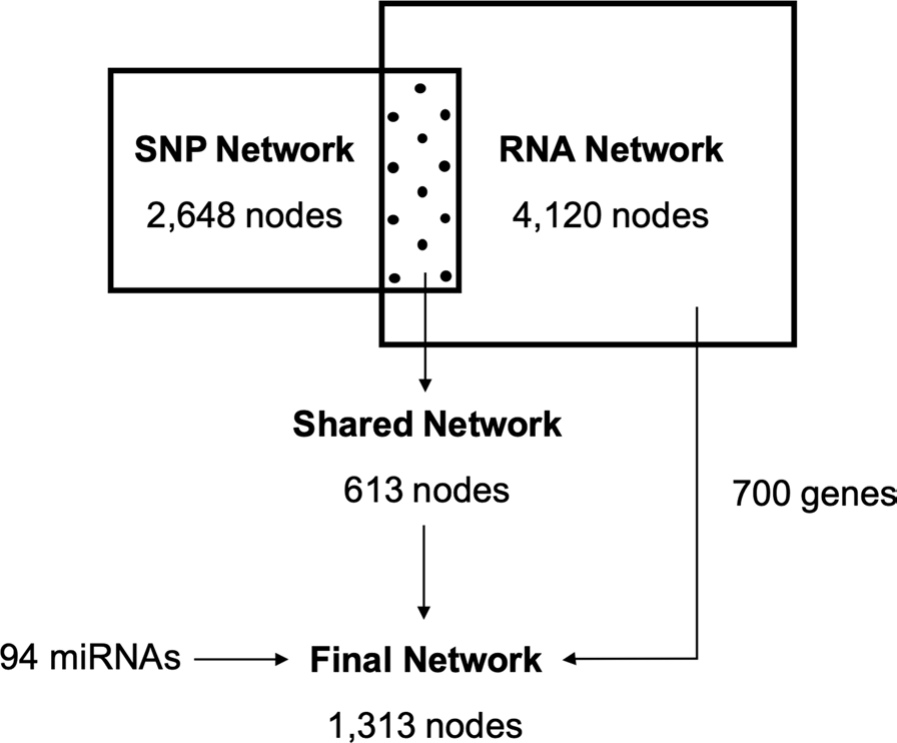
Number of nodes (genes) in each of the gene network analyses. The SNP network involved 2648 nodes connected by 2,984,616 edges (interactions). The RNA Network included 4120 nodes connected by 1,173,995 edges. The shared network included the 613 nodes and 16,591 edges present in both the SNP and the RNA networks. The final network included (i) the shared network, (ii) 700 additional genes corresponding to genes that correlated with more than four traits and their interacting genes (iii) as well as 94 co-associated miRNAs. These miRNAs interacted with 202 nodes involving 1564 edges

After SNP selection, 2648 of the 466,592 SNPs were retained to build the AWM. Trait hierarchical cluster distributions were in agreement with the biological similarities and phenotypic correlations (see Additional file 2: Figures S2 and S3). A clear separation between (i) morphological abnormalities and motility parameters and (ii) cell viability and ORT was observed based on the additive effects of the SNPs calculated in the association analysis. Consistent with previous studies [58, 59], the SNPs detected with the AWM explained 74.1% of the phenotypic variance of the key phenotype (VIAB_5). The SNP network predicted with PCIT [23] resulted in significant correlations that involved 2648 nodes (all the genes) connected by 2,984,616 edges (Fig. 2).

For the RNA network analysis, the RNA levels of the 4120 detected genes were used to identify potential connections using PCIT [23]. The RNA network included 4120 nodes (all the genes) connected by 1,173,995 edges (Fig. 2). PCIT also built 4539 significant interactions between 95 miRNAs and 630 genes.

**Fig. 3:**
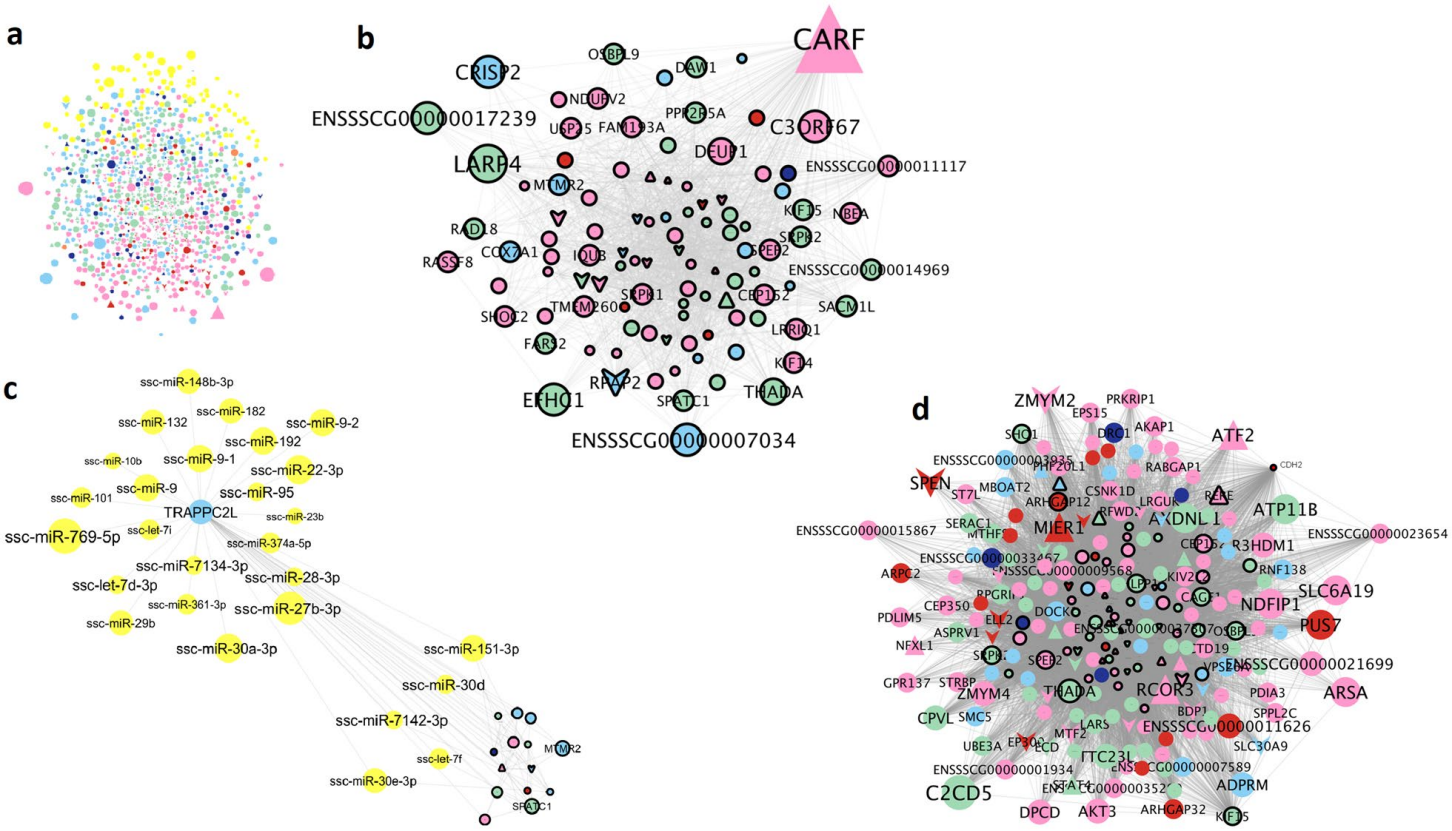
Co-association network based on the AWM and transcriptomics data. **a** Full network with 1313 genes and 94 miRNAs; **b** Subset of the network showing the transcription factor *CARF* and all its predicted interactions; **c** Subset of the network with the *TRAPPC2L* interactions, which included several miRNAs; **d** Subset of the network with the *CHD2* gene interactions. The node color corresponds to the phenotype group with the highest correlation value, as follows: concentration (red), abnormal acrosomes (green), abnormalities and droplets (pink), osmotic resistance test (orange), motility (light blue) and viability (dark blue). miRNAs are depicted in yellow. Node and text sizes correspond to the number of significant phenotypes correlated with that gene or miRNA. Nodes with a black line border correspond to genes identified in the shared network. Node shape indicates classification as: triangle (TF), V (TF co-factor) and ellipse (other genes and miRNAs)

To obtain the shared network, common SNP and RNA network edges were extracted, thus, focusing only on the shared set of interacting genes from both approaches. This comparison resulted in 613 nodes connected by 16,591 edges (Fig. 2). The final network included a set of 344 additional genes (since they correlated with more than three phenotypes) and their interactions, resulting in 700 genes. Moreover, the final network also involved 1564 edges connecting 202 genes and 94 miRNAs (Fig. 2). Of the 1313 genes included in the final network, 1135 had an abundance that correlated with at least one phenotype, 68 have been reported as TF and 89 as TF co-factors (Fig. 3a). Nearly a quarter of the genes (282 of the 1313) presented at least 200 edges. The genes that presented the largest number of interactions were *PLCH2* (579 edges, present in the final but not in the shared network and correlated with three phenotypes), *CEP152* (399 edges, in the shared network and correlated with four traits) and *SLC41A2* (382 edges, in the shared network).

Gene ontology analysis of the genes included in the final network presented enrichment for DNA repair (e.g. *RAD51*, *SETX*, and *SOD1*), meiotic cell cycle (e.g*. BAG6*, *HSPA2*, and *RAD51*), gamete generation (e.g. *TSSK3*, *PRDM14*, and *PRKAR1A*) and spermatogenesis (e.g. *BAG6*, *CAPZA3*, and *HSPA2*) (see Additional file 11: Table S9).

### Development of an RNA model and a SNP panel

The R^2^ model predicted that the RNA levels of 20 genes could explain between 55 to 78% of the phenotypic variation across traits. The 10 selected genes that were most commonly present in all the phenotype models explained the vast majority (93 to 99%) of the phenotypic variation that was predicted by the model. The final set of 10 genes included in the linear regression model was composed of *MICAL3*, *EFHC1*, *TRAPPC2L*, *ATP9A*, *THADA*, *MOBKL3*, *BLVRB*, *LARP4*, *CARS2*, and *NDUFV2.* The analysis resulted in significant models for 10 of the 25 phenotypes (Table 5). The most significant model was for PDROP, which could predict the phenotype with an efficiency of 68% (Table 5). The estimated parameters of the significant models are in Additional file 12: Table S10.

**Table 5:** R^2^ and phenotypic variance for each trait from the RNA model and SNP panel

Acronym	RNA model		SNP panel
	R^2^	P-value	Phenotypic variance explained (SE)
CON	0.17	0.82	0.05 (0.05)
VIAB_5	0.43	0.06	0.27 (0.07)
VIAB_90	0.23	0.61	0.28 (0.07)
ORT	0.22	0.62	0.24 (0.07)
HABN	0.16	0.84	0.29 (0.06)
NABN	0.22	0.64	0.36 (0.07)
TABN	0.26	0.49	0.26 (0.07)
PDROP	0.68	< 0.0001	0.17 (0.07)
DDROP	0.42	0.07	0.06 (0.05)
MT_5	0.46	0.03	0.31 (0.07)
MT_90	0.34	0.22	0.30 (0.07)
VAP_5	0.58	0.002	0.34 (0.07)
VAP_90	0.55	0.005	0.34 (0.07)
VCL_5	0.61	0.001	0.33 (0.07)
VCL_90	0.55	0.01	0.34 (0.07)
VSL_5	0.36	0.16	0.31 (0.07)
VSL_90	0.61	0.001	0.33 (0.07)
ACRO_5	0.5	0.02	0.21 (0.06)
ACRO_90	0.21	0.68	0.23 (0.07)
R_MT	0.3	0.35	0.13 (0.06)
R_VAP	0.18	0.79	0.18 (0.07)
R_VCL	0.28	0.42	0.14 (0.07)
R_VSL	0.21	0.68	0.21 (0.07)
R_VIAB	0.44	0.05	0.23 (0.07)
R_ACRO	0.57	0.003	0.19 (0.07)

Acronym descriptions are in Table 1

*SE* standard error

The SNP-based panel was built with 73 SNPs (18 lead SNPs from GWAS hits, 2 lead SNPs from the eGWAS hits, 53 SNPs from the shared network and correlated ≥ 4 phenotypes) (see Additional file 13: Table S11). These polymorphisms could explain between 5 and 36% of the phenotypic variance across the 25 traits (Table 5). A moderate proportion (> 20%) of the phenotypic variance could be explained for 18 of the 25 traits. The best predictions were for sperm abnormalities (NABN, HABN, TABN) and sperm motility related traits (e.g. MT_5, VAP_90 and VCL_90) (Table 5).

## Discussion

Investigating the genomic regions and molecular processes that control sperm quality has become a focus of interest in humans and in livestock including swine, in the latter case for its relevance on the sustainability of pig breeding and production [5, 6, 10–12]. In fact, our results and those obtained by other groups [5–8], have shown that boar sperm quality has a genetic basis, which means that it can be selected for in breeding strategies. Here, we provide an exploratory analysis using multiple bioinformatics tools. Since our study was carried out on a relatively small sample size with one phenotypic ejaculate evaluated per boar, and sperm quality traits are influenced to a great extent by environmental factors [60], our results should be considered as preliminary. This is the first study that explores at the genomic level the molecular components of sperm and semen quality using an integrative approach that fits GWAS and RNA-seq data. Moreover, our study includes, for the first time, traits such as ORT, ACRO, VIAB and the dissection of morphological abnormalities of different parts of the sperm cell (HABN, NABN and TABN).

### GWAS analysis

The GWAS revealed 12 QTL that were represented by two or more significant SNPs and several positional candidate genes for HABN, NABN, ACRO_5 and MT_5 (Table 2). The highest signals were observed on SSC4 for ACRO_5 (~ 2.41–2.42 Mbp) (Table 2) and (see Additional file 4: Table S2), ~ 69 kb upstream of the *solute carrier family 45 member 4* (*SLC45A4*) gene. *SLC45A4* encodes a proton-coupled sugar transporter that plays a role in the nutrition of spermatozoa during their maturation in the epididymis [61] where acrosome assembly continues during the post-testicular maturation phase [62]. Another *solute carrier family 35 member B3* (*SLC35B3)* was selected as a potential candidate for the MT_5 QTL on SSC7 (Table 2) and (see Additional file 4: Table S2). *SLC35B3* maps 0.6 Mbp away from this QTL.

We detected several significant regions for HABN (Table 2). Interestingly, HABN showed little correlation with the other phenotypes (see Additional file 4: Table S2), but the biological rationale behind this remains to be elucidated. The QTL on SSC1 I2 (~ 94.9–98.8 Mbp) included interesting candidate genes such as the *katanin catalytic subunit A1 like 2* (*KATNAL2*). Dunleavy et al. [63] reported that, in mice, *Katnal2* is a critical regulator of male germ cell development by affecting sperm head shaping, acrosome attachment and sperm tail growth. Other candidate genes in that region were *SLC14A2,* encoding the urea transporter A, which has been suggested to participate in sperm head formation by reducing its volume though excretion of urea [64], and the *SMAD family member 2* (*SMAD2*), which is involved in spermatogonial differentiation [65]. On SSC13 I1, we identified two candidate genes: the *testis and ovary-specific PAZ domain gene 1* (*TOPAZ1*) and the *IQ motif containing F1* (*IQCF1*). Luangpraseuth-Prosper et al. [66] demonstrated that *Topaz1-*knockout mice presented meiotic arrest and male infertility. As for *IQCF1,* Fang et al. [67] reported that this gene localizes in the acrosome and that it is involved in sperm capacitation in mice. *Iqcf1*^*−/−*^ mice were significantly less fertile than wild type mice [67]. The QTL region on SSC13 I2 included the candidate *protein kinase C delta* (*PRKCD*) gene. *PRKCD* is involved in spermatogenesis and embryonic development [68] and was highlighted in a GWAS for semen volume in Holstein–Friesian bulls [69].

Four QTL regions were identified for NABN (Table 2) and (see Additional file 4: Table S2). The QTL on SSC1 I5 included as candidate gene the transporter *ATP binding cassette subfamily A member 1* (*ABCA1*). In humans, ABCA1 localizes on the dorsal side of the sperm head and in the middle piece of the tail [70]. ABCA1 has been suggested to contribute to cholesterol transport and fertilization capacity [70].

Four of our GWAS hits are located near previously reported QTL for semen quality traits. This is the case for the SSC1 I6 QTL, associated to NABN, which mapped 335 kbp downstream from a QTL associated to sperm abnormalities and motility in boars [6]. The QTL SSC3 I2, associated to NABN lies 350 kpb upstream from a PDROP QTL [12]. The SSC4 I1 QTL, associated to ACRO_5, resides 655 kpb upstream from a QTL for the distal midpiece reflex [12] and the SSC7 I1 QTL, associated to MT_5 maps 123 kpb upstream from a PDROP QTL [12]. These discrepancies across studies could arise due to different technical (e.g. sample size, SNP arrays, QTL or phenotyping accuracy), environmental (e.g. temperature, animal husbandry or sperm processing), or biological factors (e.g. genetic heterogeneity).

### SNP calling from RNA-seq data

Calling genomic variants from RNA-seq data can be a complementary method to detect previously unknown or ungenotyped polymorphisms in transcribed genes that might carry important functional implications or may be better genetic markers for that given trait. Should these genes be involved in related phenotypes and should the variants be: (i) in LD with the GWAS lead SNP and (ii) have a predicted effect on protein sequence, these polymorphisms could be suggested as potential causal candidates. For that purpose, we sought to identify transcribed variants in the QTL regions and assessed their LD with the lead SNP hit of the QTL. Having said that, RNA-seq has some particular characteristics (namely splicing that makes read alignment challenging, allele specific expression that could miscall a true heterozygous animal with an incorrect homozygous genotype and RNA editing that post-transcriptionally generates additional variation not present in the DNA sequence), which make genotype calling from RNA-seq a challenging task. Moreover, our analysis was carried on a small number of samples (N = 35). Consequently, although we used stringent criteria for genotype calling, these results should be considered as merely indicative and a larger number of samples should be analysed to draw more robust conclusions.

For HABN, we found new genetic variants in genes of physiological interest (Table 3) and (see Additional file 7: Table S5). On SSC13 I1, we detected several variants in the *unc-51 like kinase* 4 (*ULK4*) gene, which is in potential LD with the lead SNP of this GWAS hit (Table 3) and (see Additional file 7: Table S5). Although *ULK4* has not been directly linked to sperm traits, Liu et al. [71] showed that this gene has an essential role in ciliogenesis, a process that is also crucial in sperm. The previously discussed GWAS positional and physiological candidate genes *CHD2* and *KATNAL2*, also presented genetic variants in putative LD with the lead SNPs on SSC7 I2 (low effects: rs330912302 LD = 0.4 and rs339719658 LD = 0.37) and SSC1 I3 (low effects: rs700749617 LD = 0.01, rs710447566 LD = 0.07 or moderate effect: rs690151450 LD = 6.9 × 10^–3^), respectively (see Additional file 7: Table S5). Although the results on SNP calling and LD evaluation should be taken with caution, these SNPs in *CHD2* and *KATNAL2* deserve further investigation in larger datasets.

### The porcine sperm transcriptome

The transcriptome profile obtained in this study is very similar to that from our previous work [13] and from research in other species [72, 73]. Five of the 10 most abundant protein-coding transcripts (*PRM1*, *OAZ3*, *DNAJB8*, *TPPP2* and *TNP1*) have been associated to sperm function via different mechanisms. PRM1 is a protamine that replaces histones in the ultra-compacted chromatin of sperm. In a study on bulls, the RNA levels of *PRM1* were reduced in low-fertility animals [74], and in humans, PRM1/PRM2 sperm ratios differed between fertile and infertile men [75]. OAZ3 plays a role in the regulation of polyamine concentration during spermiogenesis and has been linked to sperm function and fertility in different species such as humans [76] and mice [77]. DNAJB8 is a heat shock binding protein that regulates the ATPase activity of HSP70, which is a crucial protein for male fertility and spermatogenesis, and it shows reduced RNA levels in infertile men [78]. TPPP2 has been shown to affect sperm motility, probably by regulating energy production, and fertility in mice [79]. TNP1 is a spermatid specific protein that is involved in the replacement of histones by protamines in the sperm chromatin [80] and defects in this gene have been shown to cause male infertility [80].

Our sperm samples contained also a large and varied population of piRNAs (see Additional file 5: Table S3) [20] and, to a lesser extent, of miRNAs (see Additional file 5: Table S3 and Additional file 6: Table S4). piRNAs play an essential role in transposon silencing, are crucial for proper spermatogenesis [2], and have been annotated in the sperm of multiple animals species including humans [81], mice [82], bull [15] and boar [20]. In this study, we focused on the miRNA fraction since, as reviewed by Noora Kotaja [83], their involvement in the maintenance and regulation of spermatogonial stem cell, meiotic and post-meiotic processes and spermiogenesis is well documented. Some of the most abundant miRNAs identified in our dataset (see Additional file 6: Table S4) present relevant functions for spermatogenesis and embryo development. As a matter of fact, six of the seven miRNA with an average abundance CPM higher than 1000 have been linked to sperm function or male fertility. For example, miR-34c has been proposed to be essential for spermatogenesis, since its absence leads to infertility in mouse [84], miR-30c that is upregulated in high motile bull spermatozoa [15], and miR-191, have abundance levels that are significantly correlated with improved human embryo development [85]. Let-7 has been suggested as a regulator of IGF1 during the differentiation of spermatogonia to primary spermatocytes [86]. Recently, the level of miR-425-5p in the boar sperm has been linked to farrowing rate and litter size [87].

### Correlation between genes and miRNAs with semen traits

For mRNA transcripts, the strongest correlation was between *TTC28* and HABN (− 0.71) (see Additional file 8: Table S6). TTC28 is required for the condensation of spindle microtubules during mitosis and meiosis [88]. Other genes of interest included *ABCA3*, its RNA levels correlating with nine phenotypes (see Additional file 8: Table S6). This gene encodes an ABC transporter that plays a role in flipin-cholesterol complexes as a mechanism to remove cholesterol from the sperm membrane [89]. Although the molecular basis induced by cholesterol efflux from sperm is not well understood, it has been reported to be required for sperm capacitation [90]. Another example is *EFHC1* with RNA levels that correlated with six phenotypes (see Additional file 8: Table S6). E*fhc1*^*−/−*^ knockout mice show a reduced flagellar beating frequency [91].

Several miRNAs of interest including miR-23a, miR-27a and miR-122 correlated with seven, eight and eight semen quality traits, respectively (see Additional file 9: Table S7). miR-23a, is dysregulated in subfertile men [92], abundance of miR-27a in spermatozoa is associated with lower progressive motility and normal morphology [93], and expression of miR-122 is associated with abnormal sperm development [94] and dysregulated in subfertile men [95].

### eGWAS

GWAS hit SNPs may tag causal variants with regulatory functions on gene expression. For this reason, we also performed a within-trait eGWAS by linking for each phenotype, GWAS lead SNPs with genes that have RNA abundance correlated with the same trait. A robust eGWAS would require a larger sample size. However, we considered that the analysis was worthwhile as it could provide indicative results, which would deserve further investigation in larger populations. We identified three eQTL all with a *trans-*effect (Table 4) and (see Additional file 10: Table S8). Only one of these regions included genes of interest that were directly associated to sperm quality, i.e. the *trans*-eQTL on SSC13 for HABN, which was associated to several genes including *actin related protein 2* (*ACTR2*) and *histidyl-TRNA synthetase* (*HARS*) (Table 4) and (see Additional file 10: Table S8). Heid et al. [96] identified ACTR2 in the sperm head from bulls and suggested that it has a role in sperm capacitation and acrosome reaction. HARS has also been reported to be involved in the attachment of histidines to their corresponding tRNA molecules, a fundamental cellular process for the translation of mRNA into protein [97]. Waldron et al. [98] showed that knockout zebrafish for *HARS* presented severe defects in high proliferative cells. Although its role in sperm remains unknown, HARS is overexpressed in sperm of low-fertility bulls [99] and we do not rule out a potential involvement of this gene in spermatogenesis. *trans*-eQTL hotspots (these trans-eQTL involving several genes) are of particular interest since their SNPs could have important regulatory roles and influence gene expression, and thus are more likely to contribute to the phenotype.

### Gene network analysis

In spite of the considerable number of candidate genes that were identified in our GWAS, many genes might have been missed by this traditional single-trait approach due to the lack of an acceptable significant association (FDR > 0.05). After all, sperm quality is a complex polygenic phenotype, which is also influenced by environmental factors such as husbandry, weather, or testicular pathologies that involve an intricate network of genes and molecular processes. Moreover, low allelic frequency and low LD of the GWAS SNP with the causal variant decrease the power of the GWAS to detect genetic associations. For this reason, an alternative strategy to exploit GWAS information is to perform an AWM analysis that extracts SNPs, which although they have a strong genetic association but lower than the significance threshold, are also associated to a certain number of traits [22]. The association of one SNP to more than one trait provides additional robustness to the potential relevance of that SNP to semen quality, in our case. This, followed by a PCIT analysis to study gene–gene interactions can provide information on the relevant genes and pathways for some phenotypes and then one can search for SNPs in these genes or that affect them. Obviously, in parallel to GWAS, transcriptomics data can contribute additional valuable information in the description of these genes and pathways. The integration of both sources of information can also be used to improve the accuracy of genomic predictions [35]. We believe that the small sample size and the limitation due to measuring only one ejaculate per boar in our study can be overcome partially by the AWM and PCIT approaches. For this reason, we addressed the genetics that underlie boar sperm quality through this integrative systems biology approach. The genetic co-association and RNA co-abundance interactions revealed a number of appealing features such as new candidate genes, TF, TF co-factors, and miRNAs that belong to biological processes and relevant functions related to sperm.

The TF with the largest number of predicted interactions (129) was encoded by the *calcium responsive transcription factor* (*CARF*) gene, its RNA abundance being in turn, correlated with nine phenotypes (Fig. 3b) and (see Additional file 8: Table S6). CARF acts as a transcriptional activator promoted by calcium influx [100]. Since calcium ions are essential in sperm function [101], we cannot discard the possibility that this TF could be involved in pathways related to sperm maintenance and function. Some of the *CARF* predicted target genes from our analysis include interesting candidates such as the *la ribonucleoprotein domain family member 4* (*LARP4*), *THADA armadillo repeat containing* (*THADA*) and *EF-hand domain containing 1* (*EFHC1*) genes. *LARP4*, has been proposed to regulate mRNA stability and translation of mRNAs [102]. Blagden et al. [102] reported *larp-*knockout Drosophila mutants in which a considerable proportion of the spermatocytes had meiotic defects. Although the role of *THADA* remains uncertain in sperm, Moraru et al. [103] showed that in Drosophila*,* THADA modulates the calcium signalling, energy storage and thermogenesis balance. *EFHC1* encodes a myoclonin1 protein, which has been detected in sperm flagella in mice testis [104]. Although *Efhc1*-deficient mice were fertile, mutants presented a reduced ciliary (flagellar) beating frequency [91].

Other TF with a large number of interactions were the *SMAD family member 4* (*SMAD4*) gene (interacting with 32 genes) and the *lysine demethylase 3A* (*KDM3A*) gene (281 gene interactions), both potentially targeting a set of genes that are enriched for cellular macromolecular complex assembly processes (see Additional file 11: Table S9). TF involved in DNA repair, such as that encoded by *bromodomain adjacent to zinc finger domain 1B* (*BAZ1B*), were also identified. Its closest paralog, *BAZ1A* encodes a member of the chromatin remodeling complex [105]. Dowdle et al. [106] showed that *Baz1a*^*−/−*^ mice were infertile because of spermatogenesis defects tied to changes in chromatin composition. Another TF gene of interest was *estrogen receptor 1* (*ESR1*), which was present in the shared network. *ESR1* has already been associated with pig sperm motility and cytoplasmatic droplets [107]. Moreover, polymorphisms in *ESR1* have been suggested to influence estrogen levels which in turn, affect sperm motility [108].

The network comprised several new candidate genes for sperm quality. The *trafficking protein particle complex 2 like* (*TRAPPC2L*) gene correlated with 27 miRNAs including miR-30d, which was the most abundant miRNA in our samples (see Additional file 6: Table S4) and was found to be dysregulated in oligozoospermic men [109] (Fig. 3c). *TRAPPC2L* belongs to the TRAPPC gene family, with a reported role in ciliogenesis [110]. Interestingly, *TRAPPC2L* was associated in the final network with the *spermatogenesis and centriole associated 1* (*SPATC1*) gene, which is localized in the neck region of mouse and human sperm [111]. Disruption of its homolog *Spatc1l* in mice led to male sterility due to separation of sperm heads from tails, thereby advocating for a role in sperm head–tail integrity [112]. The network also included *DNAI2*, which correlated with four phenotypes (see Additional file 8: Table S6). Mutations in *DNAI2* have been associated with ciliary defects and detected in males with reduced fertility due to impaired sperm tail function [113]. *DNAI2* was also associated to boar sperm motility in a previous GWAS [6]. *CHD2* is another interesting gene in the network since it was also identified as a candidate gene in our GWAS analysis. This gene included new DNA variants in potential LD with GWAS lead SNPs, which would be worth testing in a genetic association study (Fig. 3d; Table 3). CHD2 was hydroxymethylated in human sperm after exposure to bisphenol A, an epigenetic modifier that causes spermatogenesis defects and alters sperm motility [114].

Of the 94 miRNAs identified in sperm and included in the final network, 30 interacted with at least 20 genes. Some of these 30 miRNAs correlated with sperm traits and have also been linked to sperm quality and fertility in previous studies. It is worth noting that miR-16, a miRNA that was found to be down-regulated in the semen of infertile males with sperm abnormalities [115], correlated with four sperm phenotypes (see Additional file 9: Table S7) and potentially interacted with 67 genes (e.g. *ATP9A*, found in the shared network and included in the RNA model). Similarly, miR-10b, previously associated with human infertile semen samples [116], correlated with a motility-related parameter (VCL) and interacted with 32 genes (including the previously discussed *TRAPPC2L* that is present in the final network).

### Development of an RNA model and a SNP panel

In this study, we provide a novel and innovative approach to develop an RNA model to estimate the phenotypes based on gene abundances. The model, which includes 10 genes, was predicted to be significant for 10 phenotypes and performed best for PDROP and some of the motility-related traits in our samples (Table 5). The model for PDROP reported a highly significant role of the *THADA* gene (see Additional file 12: Table S10), which was also present in the shared network, and its RNA levels are positively correlated with PDROP. THADA regulates energy metabolism via calcium signalling by binding the sarco/ER Ca^2+^ ATPase transporter mechanism [103] which plays an important role in the control of sperm motility acrosome reaction [117]. The *CARS2* gene was another strong contributor in the model for PDROP and was also identified in the shared network (see Additional file 12: Table S10). *CARS2* plays a critical role in protein synthesis but no direct link to spermatogenesis or sperm function has been reported.

Although SNPs have become the marker of choice for the genetic improvement of livestock species, the development of a SNP array for the prediction of boar sperm quality remains to be done. Here, we propose a SNP model with 73 SNPs including those identified through the GWAS, eGWAS and gene:gene interaction and phenotypic correlation analysis (see Additional file 13: Table S11). The model could hold promising potential for its application in animal breeding programs. This panel of 73 SNPs estimated between 5 and 36% of the phenotypic variance across the 25 traits that were evaluated. These SNPs were better predictors for the phenotypes related to sperm abnormalities and motility (Table 5). Remarkably, when considering only the GWAS lead SNPs, the panel explained between 4 and 26% of the phenotypic variance, and only for three traits (HABN, NABN and TABN) was the model able to predict more than 20% of the phenotypic variance. Thus, this systems biology approach allowed us to include an additional set of SNPs that increased the predictive potential of the panel.

In a previous study for sperm motility and morphological abnormalities using two porcine lines, Marques et al. [6] identified several QTL that cumulatively explained 10.8% of the genetic variance including 412 and 271 SNPs for each line. Gao et al. [11] identified 20 and 16 QTL that could explain 35.3 and 20.6% of sperm motility and morphological abnormalities traits in Duroc boars, respectively. Our approach was able to predict 30 to 31% and 26 to 36% of the variance of the same group of traits with only 73 SNPs for motility and morphological-related traits, respectively (Table 5). However, we have used an integrated and informed approach based not only on the GWAS and eGWAS FDR significant associations but also on a robust network built from co-associated SNPs (identified at suggestive levels but across several phenotypes) as well as gene RNA co-abundance. Moreover, our SNPs were chosen to minimize LD between them and thus maximize the informativity of the panel. This allowed the informed inclusion of a large number of SNPs with independent marker potential and thus the development of a more powerful panel for the prediction of semen quality in pigs.

These results only hold in our Pietrain population with a modest sample size and one ejaculate measured per boar, thus the validation of the panel will require additional evaluations in other populations. Nonetheless, the integrative approach that we propose for ultimately building a SNP array provides compelling results of its application to any type of complex trait with a genetic basis. This opens another avenue to improve traits that are influenced by several genes that are of interest for the animal breeding industry.

## Conclusions

In summary, our results suggest that the genetic variants identified in the 12 QTL regions that are mapped to—or near—the *CHD2*, *KATNAL2*, *SLC14A2*, *IQCF1* and *ABCA1* genes, together with other candidate genes based on a systems biology approach including among others, *LAPR4*, *THADA*, *EFHC1*, *SMADA4*, *SPATC1* or *TRAPPC2L,* may modulate sperm quality in pigs. This network also includes TF genes such as *CARF*, with a large number of potential interactions with target genes that are likely to be key players in shaping the complex inheritance of sperm quality traits. We have developed a SNP panel based on a systems biology approach that may be able to explain a larger amount of phenotypic variance than that obtained from a stand-alone GWAS. The model included GWAS lead SNPs, top eGWAS SNPs and SNPs from genes identified in the shared network and could potentially explain more than 30% of the phenotypic variance for sperm quality traits such as motility and morphology. Although our results are very promising for the pig breeding sector, caution should be taken due to the sample size of our study and the lack of repeated measures from multiple ejaculates per boar. Future work should include the validation of the RNA and SNP model in a large number of pigs belonging to different breeds and populations. The implications of this research are broad, ranging from applications to animal breeding strategies to modelling the biology of infertility in mammals.

## Supplementary Information

**Additional file 1: Figure S1.** Outline of the analysis pipeline. It illustrates the framework of the dataset, analyses and methodologies included in the study.

**Additional file 2: Figure S2.** Correlation across boar sperm quality traits. Heatmap plot of the correlations among the 25 sperm traits measured on 300 boars. CON = concentration; VIAB_5 = viability 5 min; VIAB_90 = viability 90 min; ORT = osmotic resistance test; HABN = head abnormalities; NABN = neck abnormalities; TABN = tail abnormalities; PDROP = proximal droplets; DDROP = distal droplets; MT_5 = motility 5 min; VAP_5 = average path velocity 5 min; VCL_5 = curvilinear velocity 5 min; VSL_5 = straight line velocity 5 min; MT_90 = motility 90 min; VAP_90 = average path velocity 90 min; VCL_90 = curvilinear velocity 90 min; VSL_90 = straight line velocity 90 min; ACRO_5 = abnormal acrosomes 5 min; ACRO_90 = abnormal acrosomes 90 min; R_MT = ratio motility; R_VAP = ratio average path velocity; R_VCL = ratio curvilinear velocity; R_VSL = ratio straight line velocity; R_VIAB = ratio viability; R_ACRO = ratio acrosomes. **Figure S3.** SNP based dendrogram for the 25 semen parameters. Dendrogram of the standardized SNP effects across the 25 sperm traits.

**Additional file 3: Table S1.** Effect of external factors on sperm quality traits. Effect of farm, age and season per year across the sperm quality related phenotypes. * = P-value ≤ 0.05; ** = P-value ≤ 0.001; *** = P-value ≤ 0.0001; ns = not significant.

**Additional file 4: Table S2.** Details on the SNPs showing significant associations (FDR ≤ 0.05) in the GWAS across autosomal chromosomes and unplaced scaffolds. Chr = chromosome; BP = base pairs (location); Beta = additive effect; MAF = minor allele frequency; FDR = false discovery rate; HABN = head abnormalities; MT_5 = percentage of motile spermatozoa at 5 min; MT_90 = percentage of motile spermatozoa at 90 min; NABN = neck abnormalities; PDROP = proximal droplets; R_ACRO = ratio abnormal acrosomes.

**Additional file 5: Table S3.** Details of the RNA-seq extraction and mapping statistics. Average and Standard Deviation (SD) for the 40 samples processed, including the amount of RNA obtained and several bioinformatics statistics for total RNA-seq (40 samples) and short RNA-seq (34 samples) datasets. sncRNA = short noncoding RNA; MttRNA = mitochondrial transfer RNA; piRNA = Piwi interacting RNA; snRNA = small nuclear RNA; MtrRNA = mitochondrial ribosomal RNA; tRNA = transfer RNA; miRNA = micro RNA; rRNA = ribosomal RNA; snoRNA = small nucleolar RNA.

**Additional file 6: Table S4.** List of protein coding genes and miRNAs identified in sperm. Average and Standard Deviation (SD) for the samples processed. Protein coding and miRNA abundances are expressed in fragments per kb per million mapped reads (FPKM) and counts per million (CPM), respectively.

**Additional file 7: Table S5.** SNPs identified in the RNA-seq data mapping within the GWAS regions. Chr = chromosome. LD = linkage disequilibrium. Genotypic frequency for each of the genotypes. # samples called = number of samples with reads in the given SNP position.

**Additional file 8: Table S6.** Correlations between gene abundances and phenotypes. P-values are given when (P-value ≤ 0.05). The correlation value is indicated between brackets. ns = not significant.

**Additional file 9: Table S7.** Correlations between miRNA abundances and phenotypes. P-values are given when (P-value ≤ 0.05). The correlation value is indicated between brackets. ns = not significant.

**Additional file 10: Table S8.** Associations identified in the within trait eGWAS. Thirty-nine SNPs showed significant associations (FDR ≤ 0.05) with semen phenotypes in the GWAS and also displayed significant association with the abundance of genes which abundance correlated with the same phenotype (P-value ≤ 0.05). Chr = chromosome. FDR = False Discovery Rate; ACRO_5 = Abnormal Acrosomes 5 min; HABN = Head abnormalities.

**Additional file 11: Table S9.** Gene Ontology analysis of the genes included in the Final Network. GO biological process terms with significant Bonferroni corrected P-values and their associated genes.

**Additional file 12: Table S10.** Parameter estimates for the significant RNA models. For each of the phenotypes, the model outputs the estimated values for the 10 genes obtained from the GRM regression analysis. The lower the value of Pr >|t|, the higher the involvement of the gene abundance on the total phenotypic variance.

**Additional file 13: Table S11.** Description of the SNPs included in the SNP panel. Chromosome, position, SNP ID and analysis from which the SNP was extracted.

## Data Availability

The datasets generated and/or analysed during the current study are available at NCBI’s BioProject PRJNA520978. The phenotypic and genotypic datasets used in the current study are available from the corresponding author on reasonable request.
