# Supplementary figures and images for: A systems biology framework integrating GWAS and RNA-seq to shed light on the molecular basis of sperm quality in swine
Source: Genet Sel Evol. 2020 Dec 8;52:72. doi: 10.1186/s12711-020-00592-0 (PMC7724732; doi:10.1186/s12711-020-00592-0)

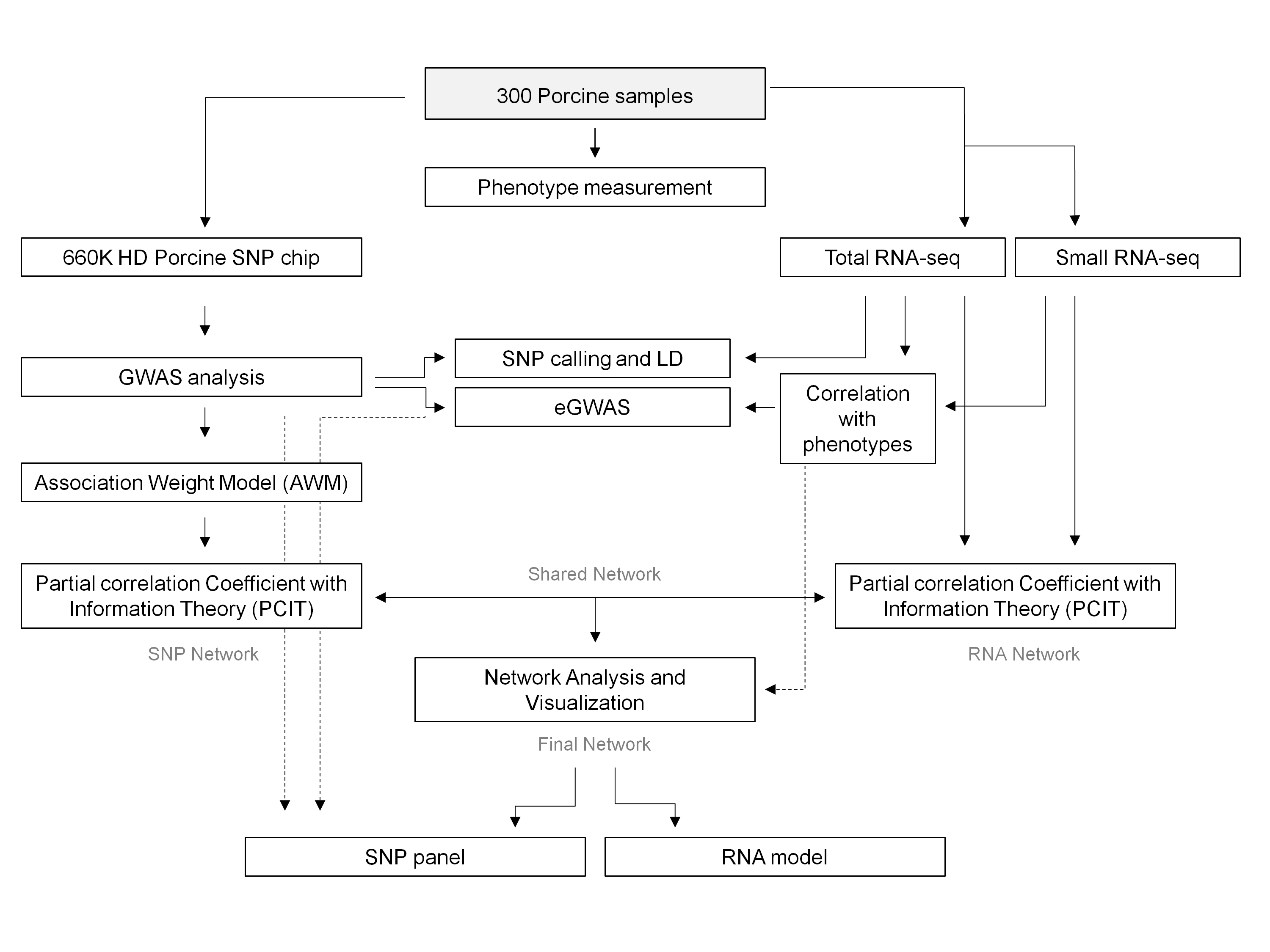

Supplement: Supplementary file 1 — Additional file 1: Figure S1. Outline of the analysis pipeline. It illustrates the framework of the dataset, analyses and methodologies included in the study. [file 12711_2020_592_MOESM1_ESM.tif]

Additional Figure S2.


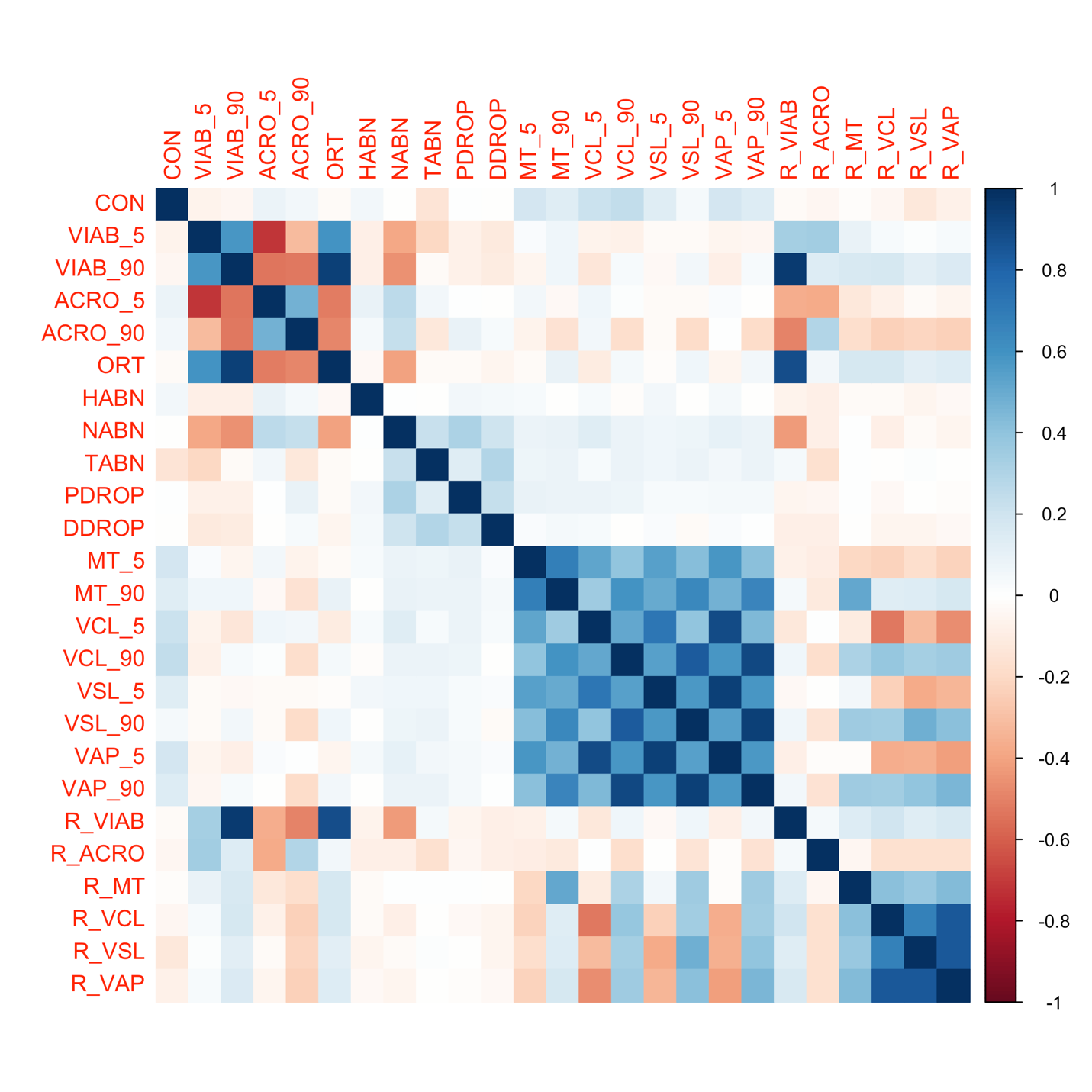


Additional Figure S3.
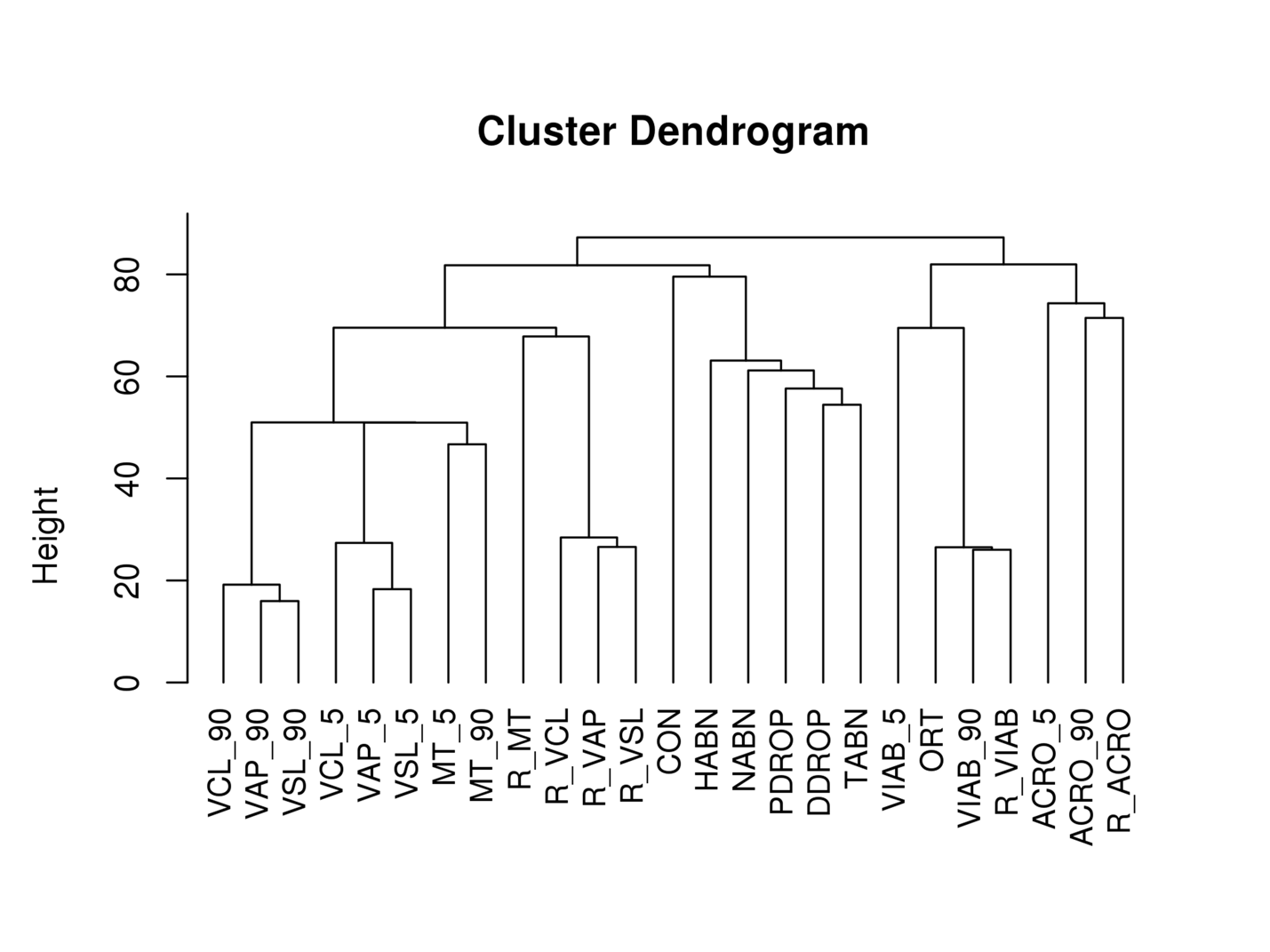

Supplement: Supplementary file 2 — Additional file 2: Figure S2. Correlation across boar sperm quality traits. Heatmap plot of the correlations among the 25 sperm traits measured on 300 boars. CON = concentration; VIAB_5 = viability 5 min; VIAB_90 = viability 90 min; ORT = osmotic resistance test; HABN = head abnormalities; NABN = neck abnormalities; TABN = tail abnormalities; PDROP = proximal droplets; DDROP = distal droplets; MT_5 = motility 5 min; VAP_5 = average path velocity 5 min; VCL_5 = curvilinear velocity 5 min; VSL_5 = straight line velocity 5 min; MT_90 = motility 90 min; VAP_90 = average path velocity 90 min; VCL_90 = curvilinear velocity 90 min; VSL_90 = straight line velocity 90 min; ACRO_5 = abnormal acrosomes 5 min; ACRO_90 = abnormal acrosomes 90 min; R_MT = ratio motility; R_VAP = ratio average path velocity; R_VCL = ratio curvilinear velocity; R_VSL = ratio straight line velocity; R_VIAB = ratio viability; R_ACRO = ratio acrosomes. Figure S3. SNP based dendrogram for the 25 semen parameters. Dendrogram of the standardized SNP effects across the 25 sperm traits. [file 12711_2020_592_MOESM2_ESM.docx]
