# Supplementary material for: A systems biology framework integrating GWAS and RNA-seq to shed light on the molecular basis of sperm quality in swine
Source: Genet Sel Evol. 2020 Dec 8;52:72. doi: 10.1186/s12711-020-00592-0 (PMC7724732; doi:10.1186/s12711-020-00592-0)
Supplement: Supplementary file 12 — Additional file 12: Table S10. Parameter estimates for the significant RNA models. For each of the phenotypes, the model outputs the estimated values for the 10 genes obtained from the GRM regression analysis. The lower the value of Pr >|t|, the higher the involvement of the gene abundance on the total phenotypic variance. [file 12711_2020_592_MOESM12_ESM.pdf]

Dependent Variable: PDROP

Analysis of Variance		
**Source**	**Pr > F**	**R-Square**
**Model**	<.0001	0.6789

Parameter Estimates					
**Variable**	**DF**	**Parameter Estimate**	**Standard Error**	**t Value**	**Pr > |t|**
**Intercept**	1	-6.67543	22.32205	-0.30	0.7671
**MICAL3**	1	-2.13027	1.25069	-1.70	0.0996
**EFHC1**	1	-3.08434	2.56321	-1.20	0.2389
**TRAPPC2L**	1	2.91347	1.80485	1.61	0.1177
**ATP9A**	1	-0.37478	1.74263	-0.22	0.8313
**THADA**	1	10.30843	3.26312	3.16	0.0038
**MOBKL3**	1	-4.10257	1.61153	-2.55	0.0167
**BLVRB**	1	-1.28970	1.61326	-0.80	0.4308
**LARP4**	1	10.19089	4.33868	2.35	0.0261
**CARS2**	1	-10.84193	2.56759	-4.22	0.0002
**NDUFV2**	1	-1.06337	0.95818	-1.11	0.2765

Dependent Variable: MT_5

Analysis of Variance		
**Source**	**Pr > F**	**R-Square**
**Model**	0.0328	0.4622

Parameter Estimates					
**Variable**	**DF**	**Parameter Estimate**	**Standard Error**	**t Value**	**Pr > |t|**
**Intercept**	1	235.87139	89.55654	2.63	0.0136
**MICAL3**	1	-15.95264	5.01779	-3.18	0.0036
**EFHC1**	1	15.23666	10.28367	1.48	0.1496
**TRAPPC2L**	1	-13.81740	7.24108	-1.91	0.0667
**ATP9A**	1	-6.94742	6.99149	-0.99	0.3289

**THADA**	**1**	4.49283	13.09171	0.34	0.7340
**MOBKL3**	**1**	-5.77069	6.46550	-0.89	0.3797
**BLVRB**	**1**	-10.10955	6.47244	-1.56	0.1295
**LARP4**	**1**	-32.83936	17.40689	-1.89	0.0696
**CARS2**	**1**	15.60875	10.30121	1.52	0.1409
**NDUFV2**	**1**	0.67538	3.84422	0.18	0.8618

Dependent Variable: VAP_5

**Analysis of Variance**		
**Source**	**Pr > F**	**R-Square**
**Model**	0.0021	0.5820

**Parameter Estimates**					
**Variable**	**DF**	**Parameter Estimate**	**Standard Error**	**t Value**	**Pr > |t|**
**Intercept**	**1**	95.79398	34.17011	2.80	0.0091
**MICAL3**	**1**	-3.74807	1.91453	-1.96	0.0603
**EFHC1**	**1**	9.34483	3.92371	2.38	0.0243
**TRAPPC2L**	**1**	-5.44912	2.76282	-1.97	0.0585
**ATP9A**	**1**	-4.37134	2.66759	-1.64	0.1125
**THADA**	**1**	-2.45373	4.99511	-0.49	0.6271
**MOBKL3**	**1**	-1.62237	2.46690	-0.66	0.5161
**BLVRB**	**1**	-7.49411	2.46954	-3.03	0.0052
**LARP4**	**1**	-7.60258	6.64156	-1.14	0.2620
**CARS2**	**1**	6.05058	3.93041	1.54	0.1349
**NDUFV2**	**1**	-1.10720	1.46675	-0.75	0.4566

Dependent Variable: VCL_5

**Analysis of Variance**		
**Source**	**Pr > F**	**R-Square**
**Model**	0.0010	0.6091

**Parameter Estimates**

Variable	DF	Parameter Estimate	Standard Error	t Value	Pr > |t|
**Intercept**	**1**	119.72634	48.73351	2.46	0.0205
**MICAL3**	**1**	-6.54087	2.73050	-2.40	0.0235
**EFHC1**	**1**	13.13625	5.59601	2.35	0.0262
**TRAPPC2L**	**1**	-8.82712	3.94034	-2.24	0.0332
**ATP9A**	**1**	-8.39816	3.80452	-2.21	0.0356
**THADA**	**1**	-5.04435	7.12405	-0.71	0.4848
**MOBKL3**	**1**	-0.65897	3.51830	-0.19	0.8528
**BLVRB**	**1**	-8.69914	3.52207	-2.47	0.0199
**LARP4**	**1**	-10.57440	9.47221	-1.12	0.2738
**CARS2**	**1**	10.61561	5.60556	1.89	0.0686
**NDUFV2**	**1**	0.16945	2.09189	0.08	0.9360

Dependent Variable: VAP_90

Analysis of Variance		
**Source**	**Pr > F**	**R-Square**
**Model**	0.0045	0.5530

Parameter Estimates					
**Variable**	**DF**	**Parameter Estimate**	**Standard Error**	**t Value**	**Pr > |t|**
**Intercept**	**1**	179.85589	37.60544	4.78	<.0001
**MICAL3**	**1**	-4.72561	2.10701	-2.24	0.0330
**EFHC1**	**1**	11.04539	4.31819	2.56	0.0162
**TRAPPC2L**	**1**	-7.87030	3.04058	-2.59	0.0151
**ATP9A**	**1**	0.23718	2.93578	0.08	0.9362
**THADA**	**1**	-1.68146	5.49730	-0.31	0.7620
**MOBKL3**	**1**	-5.64055	2.71491	-2.08	0.0470
**BLVRB**	**1**	-4.25291	2.71782	-1.56	0.1289
**LARP4**	**1**	-17.81838	7.30928	-2.44	0.0214
**CARS2**	**1**	-3.77502	4.32555	-0.87	0.3902
**NDUFV2**	**1**	-0.52489	1.61422	-0.33	0.7475

Dependent Variable: VCL_90

Analysis of Variance		
**Source**	**Pr > F**	**R-Square**
**Model**	0.0054	0.5459

Parameter Estimates					
**Variable**	**DF**	**Parameter Estimate**	**Standard Error**	**t Value**	**Pr > |t|**
**Intercept**	**1**	207.96414	42.53019	4.89	<.0001
**MICAL3**	**1**	-6.07376	2.38294	-2.55	0.0166
**EFHC1**	**1**	13.73971	4.88369	2.81	0.0089
**TRAPPC2L**	**1**	-11.46167	3.43877	-3.33	0.0024
**ATP9A**	**1**	3.69441	3.32024	1.11	0.2753
**THADA**	**1**	-7.18479	6.21722	-1.16	0.2576
**MOBKL3**	**1**	-5.49314	3.07045	-1.79	0.0844
**BLVRB**	**1**	-5.75048	3.07374	-1.87	0.0719
**LARP4**	**1**	-21.21308	8.26649	-2.57	0.0159
**CARS2**	**1**	-0.73974	4.89202	-0.15	0.8809
**NDUFV2**	**1**	0.62781	1.82561	0.34	0.7335

Dependent Variable: VSL_90

Analysis of Variance		
**Source**	**Pr > F**	**R-Square**
**Model**	0.0009	0.6129

Parameter Estimates					
**Variable**	**DF**	**Parameter Estimate**	**Standard Error**	**t Value**	**Pr > |t|**
**Intercept**	**1**	160.39335	30.51670	5.26	<.0001
**MICAL3**	**1**	-4.48719	1.70983	-2.62	0.0139
**EFHC1**	**1**	9.76489	3.50419	2.79	0.0095
**TRAPPC2L**	**1**	-5.89665	2.46742	-2.39	0.0238
**ATP9A**	**1**	-0.58723	2.38237	-0.25	0.8071
**THADA**	**1**	2.43030	4.46104	0.54	0.5902

Parameter Estimates					
Variable	DF	Parameter Estimate	Standard Error	t Value	Pr > |t|
**MOBKL3**	1	-4.56517	2.20314	-2.07	0.0476
**BLVRB**	1	-3.43994	2.20550	-1.56	0.1301
**LARP4**	1	-17.39652	5.93146	-2.93	0.0066
**CARS2**	1	-5.74714	3.51017	-1.64	0.1128
**NDUFV2**	1	-0.98537	1.30993	-0.75	0.4582

Dependent Variable: ACRO_5

Analysis of Variance		
Source	Pr > F	R-Square
**Model**	0.0152	0.5008

Parameter Estimates					
Variable	DF	Parameter Estimate	Standard Error	t Value	Pr > |t|
**Intercept**	1	-35.03213	23.99593	-1.46	0.1554
**MICAL3**	1	0.51430	1.34447	0.38	0.7050
**EFHC1**	1	-2.74212	2.75542	-1.00	0.3282
**TRAPPC2L**	1	3.99231	1.94019	2.06	0.0490
**ATP9A**	1	-3.97201	1.87331	-2.12	0.0430
**THADA**	1	-2.65893	3.50781	-0.76	0.4548
**MOBKL3**	1	0.43657	1.73238	0.25	0.8029
**BLVRB**	1	1.45652	1.73424	0.84	0.4081
**LARP4**	1	11.58789	4.66403	2.48	0.0192
**CARS2**	1	-3.08530	2.76012	-1.12	0.2731
**NDUFV2**	1	-0.46316	1.03003	-0.45	0.6564

Dependent Variable: R_VIAB

Analysis of Variance		
Source	Pr > F	R-Square
**Model**	0.0489	0.4402

Parameter Estimates					
**Variable**	**DF**	**Parameter Estimate**	**Standard Error**	**t Value**	**Pr > |t|**
**Intercept**	**1**	-114.93179	87.76548	-1.31	0.2010
**MICAL3**	**1**	-6.96496	4.91744	-1.42	0.1677
**EFHC1**	**1**	1.42685	10.07800	0.14	0.8884
**TRAPPC2L**	**1**	8.40268	7.09626	1.18	0.2463
**ATP9A**	**1**	4.72811	6.85166	0.69	0.4958
**THADA**	**1**	13.93994	12.82988	1.09	0.2865
**MOBKL3**	**1**	3.17950	6.33619	0.50	0.6197
**BLVRB**	**1**	-10.86517	6.34299	-1.71	0.0978
**LARP4**	**1**	13.27225	17.05876	0.78	0.4431
**CARS2**	**1**	6.85345	10.09520	0.68	0.5028
**NDUFV2**	**1**	-6.96331	3.76734	-1.85	0.0751

Dependent Variable: R_ACRO

Analysis of Variance		
**Source**	**Pr > F**	**R-Square**
**Model**	0.0029	0.5708

Parameter Estimates					
**Variable**	**DF**	**Parameter Estimate**	**Standard Error**	**t Value**	**Pr > |t|**
**Intercept**	**1**	-89.34339	39.67014	-2.25	0.0323
**MICAL3**	**1**	-3.82073	2.22269	-1.72	0.0967
**EFHC1**	**1**	0.31082	4.55527	0.07	0.9461
**TRAPPC2L**	**1**	4.77681	3.20752	1.49	0.1476
**ATP9A**	**1**	2.15941	3.09696	0.70	0.4914
**THADA**	**1**	8.86449	5.79913	1.53	0.1376
**MOBKL3**	**1**	3.92658	2.86397	1.37	0.1813
**BLVRB**	**1**	-3.99020	2.86704	-1.39	0.1750
**LARP4**	**1**	4.89351	7.71059	0.63	0.5308
**CARS2**	**1**	5.05271	4.56305	1.11	0.2776
**NDUFV2**	**1**	-2.41878	1.70284	-1.42	0.1665
